# Differential contribution of immune effector mechanisms to cortical demyelination in multiple sclerosis

**DOI:** 10.1007/s00401-017-1706-x

**Published:** 2017-04-06

**Authors:** Nielsen Lagumersindez-Denis, Claudia Wrzos, Matthias Mack, Anne Winkler, Franziska van der Meer, Marie C. Reinert, Heiko Hollasch, Anne Flach, Hilke Brühl, Eilish Cullen, Christina Schlumbohm, Eberhard Fuchs, Christopher Linington, Alonso Barrantes-Freer, Imke Metz, Christiane Wegner, David Liebetanz, Marco Prinz, Wolfgang Brück, Christine Stadelmann, Stefan Nessler

**Affiliations:** 1 Institute of Neuropathology, University Medical Center Göttingen, 37075 Göttingen, Germany; 2 Department of Internal Medicine, University Hospital Regensburg, Regensburg, Germany; 3 Department of Clinical Neurophysiology, University Medical Center Göttingen, 37075 Göttingen, Germany; 4 Department of Paediatrics and Paediatric Neurology, University Medical Center Göttingen, 37075 Göttingen, Germany; 5 Department of Neurology, Albert Ludwigs University, Freiburg, Germany; 6 Department of Neuroimmunology, Institute for Multiple-Sclerosis-Research, University of Göttingen and Hertie Foundation, 37073 Göttingen, Germany; 7 Medical Research Council Technology, Lynton House, 7-12 Tavistock Square, London, WC1H 9LT UK; 8 Neu Encepharm GmbH, Göttingen, Germany; 9 German Primate Center, Göttingen, Germany; 10 Institute for Infection, Immunity and Inflammation, University of Glasgow, 120 University Place, Glasgow, G12 8TA UK; 11 Institute of Neuropathology, University of Freiburg, Freiburg, Germany; 12 BIOSS Centre for Biological Signalling Studies, University of Freiburg, Freiburg, Germany; 13 Klinik und Poliklinik für Neurologie and Center for Stroke Research Berlin, Charité Universitätsmedizin Berlin, 10117 Berlin, Germany

**Keywords:** Progressive multiple sclerosis, Cortical demyelination, Experimental autoimmune encephalomyelitis, Inflammatory monocytes

## Abstract

Cortical demyelination is a widely recognized hallmark of multiple sclerosis (MS) and correlate of disease progression and cognitive decline. The pathomechanisms initiating and driving gray matter damage are only incompletely understood. Here, we determined the infiltrating leukocyte subpopulations in 26 cortical demyelinated lesions of biopsied MS patients and assessed their contribution to cortical lesion formation in a newly developed mouse model. We find that conformation-specific anti-myelin antibodies contribute to cortical demyelination even in the absence of the classical complement pathway. T cells and natural killer cells are relevant for intracortical type 2 but dispensable for subpial type 3 lesions, whereas CCR2^+^ monocytes are required for both. Depleting CCR2^+^ monocytes in marmoset monkeys with experimental autoimmune encephalomyelitis using a novel humanized CCR2 targeting antibody translates into significantly less cortical demyelination and disease severity. We conclude that biologics depleting CCR2^+^ monocytes might be attractive candidates for preventing cortical lesion formation and ameliorating disease progression in MS.

**Electronic supplementary material:**

The online version of this article (doi:10.1007/s00401-017-1706-x) contains supplementary material, which is available to authorized users.

## Introduction

The cortex is a major predilection site for demyelination in multiple sclerosis (MS) [18]. Cortical pathology is increasingly recognized in all MS phenotypes by non-conventional ultra-high field magnetic resonance imaging (MRI) from the earliest disease stages on, including pediatric-onset MS [1]. The presence of gray matter damage has been associated with long-term physical and cognitive impairment [11] and has early prognostic relevance for the conversion to clinically definite MS [15]. Also, MRI studies have consistently demonstrated that gray matter atrophy reflects disability progression better than white matter atrophy or T2 lesion load, suggesting that cortical pathology plays a pivotal role in disease progression [12].

Three cortical lesion types have been distinguished in studies of MS pathology according to topography [7, 41]: Leukocortical lesions (type 1) encompassing deep cortical areas and subcortical white matter, intracortical lesions centered on intracortical microvessels (type 2) and subpial lesions extending from the pia mater into the superficial cortical layers (type 3). Subpial type 3 lesions are the most frequent and extensive cortical lesion type and more specific to MS than white matter lesions [36]. In postmortem brain tissue of patients with chronic MS, cortical demyelinated lesions in general and subpial cortical demyelinated lesions in particular are less inflammatory than demyelinated white matter lesions [3, 7, 28]. Thus, degenerative processes have been proposed to prevail in cortical pathology. This view has been challenged by biopsy studies of cortical demyelinated lesions, which were highly inflammatory [32] and by animal studies demonstrating the rapid resolution of cortical inflammation [35]. In addition, expression signatures characteristic of innate and adaptive immune activation can be found in cortical demyelinated lesions at autopsy [16] and subpial cortical demyelination was often associated with meningeal inflammation [21]. In line, a recent imaging study demonstrated focal, long-lasting leptomeningeal contrast enhancements in postcontrast T2 weighted fluid-attenuated inversion recovery (FLAIR) MRI in MS [2]. Pathogenetically, meningeal inflammatory cells might release myelino- and neurotoxic soluble mediators, which diffuse into the superficial gray matter, contributing to demyelination and the reported gradient of neuronal damage [33].

Although there is little doubt about the causative role of inflammatory processes for cortical demyelination and ensuing neuroaxonal damage, surprisingly little quantitative data is available on immune cell subpopulations in cortical demyelinated lesions and meningeal infiltrates. Even less is known about the cellular and humoral mediators contributing to cortical demyelination. Macrophages and T cells were reported to outnumber B cells in meningeal inflammation in progressive MS patients [21] and complement deposits were inconsistently detected in human autopsy specimens [9, 52]. In the present work, we set out to study quantitatively adaptive and innate immune cell populations in a cohort of patients with early cortical demyelination. Furthermore, we employed immune cell depletion and genetic manipulations in a newly developed experimental mouse model of cortical demyelination to define the immune effector mechanisms operating in cortical demyelination in vivo. We finally translated our findings into a novel, biological therapy against cortical pathology in MS.

## Materials and methods

### Patients

We screened a cohort of 740 archival CNS biopsies of patients diagnosed with inflammatory demyelinating disease between 1999 and 2016 for the presence of cortical demyelination. Biopsies were performed for diagnostic reasons to exclude tumor or infection and targeted MRI-defined white matter lesions (Table S1). The study was approved by the ethical review committee of the University Medical Center Göttingen (#19/09/10). All biopsies were stained with HE and LFB/PAS and Bielschowsky silver impregnated. Immunohistochemistry (IHC) was performed with antibodies against myelin, oligodendrocytes, adaptive and innate immune cells, neurons and axons, astrocytes, including AQP4, JC virus and Ki-67. Biopsies with cortical demyelination were in addition stained with antibodies against CCR2 and double-labelled for CCR2/CD14 and CCR2/KiM1P to identify recently invaded monocytes and tissue macrophages, and GrB/CD3 to identify natural killer (NK) cells. Demyelinating lesion activity was determined according to Brück et al. [10] and Kuhlmann et al. [27]. Lesions without myelin-laden macrophages were staged according to Schirmer et al. [47]. Primary antibodies used for IHC of human MS biopsies are listed in Table S2 and exemplary IHC of tonsillar tissue with antibodies against CD3, CD4, CD8, CD20 and CD138 is provided as Supplemental Fig. 6.

135 biopsies (18%) contained cortical tissue in addition to subcortical white matter (Figure S1). Of those, 26 patients (19%) showed cortical demyelination identified by myelin immunohistochemistry. All but two patients also harbored subcortical white matter for analysis. 20/26 patients showed white matter demyelination in addition to cortical demyelination. Intracortical (type 2) lesions were exceedingly rare in this cohort, and leukocortical (type 1) lesions were not included in the present study.

The median age of the patients at biopsy was 41 (interquartile range, 30–49), the median time to biopsy was 2 months (interquartile range, 0.5–24) and the female to male ratio was 2.3. 10/26 patients had seizures or were diagnosed with symptomatic epilepsy and 8/26 patients presented with moderate to severe neuropsychiatric symptoms (confusion, cognitive impairment, depression; Table S1). The clinical diagnosis at the time of biopsy was CIS in 16, RRMS in 8 and SPMS in 2 cases.

### Animals

C57BL/6J mice were obtained from Charles River Laboratories (Charles River). 2D2 mice (TCR^MOG^) [6], Th/+mice (IgG^MOG^, kindly provided by Tobias Litzenburger, Antonio Iglesias and Hartmut Wekerle [31]), OSE double transgenic mice (TCR^MOG^ × IgG^MOG^) [5, 26], RAG1^−/−^ mice [37], RAG2^−/−^ γc^−/−^, RAG1^−/−^ γc^-/-^, C1q^−/−^ (kindly provided by Marina Botto [8]) and OT-II [4] mice were bred at the animal facility of the University of Göttingen under SPF conditions. Th/+CCR2^−/−^, Th/+C1q^−/−^ and Th/+CD59a^−/−^ mice were generated by crossbreeding CCR2^−/−^ [29] C1q^−/−^ or CD59a^−/−^ (kindly provided by B.P. Morgan [20]) mice with Th/Th mice. Knockout animals were backcrossed against C57BL/6J for more than 12 generations and housed at a density of 2–5 animals/cage in a temperature controlled environment with a 12/12 h light/dark cycle and food and water ad libitum. If not otherwise stated, 6–10 week old mice of both sexes were randomized into groups. Adult common marmosets of both sexes (*Callithrix jacchus*) were provided by Neu Encepharm (Göttingen, Germany).

### EAE induction and scoring

2D2 animals were immunized subcutaneously (s.c.) with 200 µg MOG_35−55_ (purchased from the Institute of Medical Immunology, University Medical Center Charité, Berlin) emulsified in CFA substituted with 5 mg/ml *Mycobacterium tuberculosis* H37Ra (Difco, 231141). C57BL/6J, Th/+ and OSE mice were immunized s.c. with 100 µg recombinant rat MOG_1–125_/CFA. 300 ng/mouse pertussis toxin (PTX) (List Biological Laboratories, #180) were injected i.p. at day 0 and day 2 after immunization. Control animals were naïve or immunized with CFA only. Age- and sex-matched common marmosets were immunized s.c. with 50 µg recombinant rat MOG_1–125_ emulsified in IFA supplemented with 0.25 mg/ml *Mycobacterium butyricum* (Difco, 264010). EAE animals were scored as previously described for mice [39] and marmosets [50].

### Depletion and blocking experiments

Monocyte depletion in marmoset monkeys was initiated 14 days after immunization by twice weekly i.v. injections of 5 mg/kg DOC-2 Fr-2 (marmoset IgG1-chimeric humanized mouse anti-human CCR2 antibody). Controls received 5 mg/kg marmoset IgG1-chimeric isotype control antibody. The administration frequency was reduced to once weekly from day 28 to the end of the experiment. In mice, all depletion and blocking experiments started at disease onset. NK cells were depleted in Th/+ mice by daily i.p. injections of 300 µg of the mouse monoclonal anti-NK1.1 antibody (Clone PK136, Bio X Cell, BE0036). Control Th/+ mice received 300 µg i.p. of the isotype control antibody C1.18.4 (Clone C1.18.4, Bio X Cell, BE0085). To block the formation of the membrane attack complex (MAC) 2 µg of the BB5.1 monoclonal antibody against mouse complement component C5 (Hycult biotech, HM1073) [23] or a mouse IgG1 control antibody (BioLegend, Clone MOPC-21) was injected intracerebrally at the time point of stereotactic cytokine injection.

### Intracerebral stereotactic injections

Mice were anaesthetized i.p. by injection of ketamine/xylazine and mounted on a stereotactic device (Stoelting Co, Germany). The scalp was opened to expose the skull and a fine hole was drilled 0.1 mm caudal to the bregma and 0.2 mm lateral to the midline. A finely calibrated glass capillary was inserted into the brain (0.7 mm depth) allowing the intracerebral administration of 2 µl of a mixture composed of 50 ng TNFα (R&D Systems) and 60 ng IFNγ (R&D Systems). Immunized animals were injected on the 2nd day of disease, animals which received cell-depleting antibodies on day 3 after EAE onset. Monastral blue (Sigma-Aldrich) was added to the cytokine mixture to facilitate the identification of the lesions in the tissue.

### Adoptive transfer experiments

Spleen cells from 2D2 or OT-II mice were expanded with plate bound anti-CD3 (4 µg/ml, Bio X Cell, Clone 145-2C11, BE0001-1) and soluble anti-CD28 (1 µg/ml, Bio X Cell, Clone PV1, BE0015-5) in the presence of 1 ng/ml rm IL-12 (R&D systems). Cells were restimulated with 15 µg/ml MOG_35–55_ or 15 µg/ml chicken ovalbumin 323–339 (OVA) and 30 Gy-irradiated antigen presenting cells for 3 days and 10 million T cell blasts were injected i.p. into RAG1^−/−^, RAG1^−/−^ γc^−/−^ or RAG2^−/−^ γc^−/−^ animals. 12 h after adoptive transfer, all animals were s.c. immunized with 10 µg MOG_35–55_ or OVA peptide and received 300 ng PTX i.p. On the 2nd day of EAE 1.5 mg/animal of the MOG-specific antibodies 8-18C5 (IgG1 isotype) or Z2 (IgG2a isotype) was injected i.v., and animals were subjected to stereotactic surgery.

### Motor skill sequence (MOSS) test

Male Th/+ and C57BL/6J mice were individually equipped with a running wheel with regularly spaced crossbars (conventional wheels) where they could run freely at any time for 2 weeks [30]. After this period they were immunized s.c. with a subclinical dose of 10 µg recombinant rat MOG_1–125_/CFA, therefore, not developing clinical disease. The mice were kept on the conventional running wheels for further 11 days and then randomized according to their wheel running performance into two comparable groups which received intracortical stereotactic injections of 2 µl PBS or cytokines. One day after surgery, all animals were put onto wheels with irregularly spaced crossbars (complex wheels). Wheel running performance was recorded continuously by LabVIEW™-based software.

### Preparation of CNS mononuclear cells and flow cytometry

The meninges were removed and the cortex was separated from the white matter 2 days after stereotactic injection, cut and digested for 45 min at 37 °C with 2.5 mg/ml Collagenase D (Roche) and 1 mg/ml DNAse I (Roche). Mononuclear cells were isolated by Percoll gradient centrifugation (37%/70%, GE Healthcare) removed from the interphase, washed and subsequently blocked with αCD16/32 (BioLegend, Clone 93) for 15 min. The following antibodies were used (all from BioLegend or eBioscience): αCD3 (145-2C11), αCD4 (RM4-5), αCD8 (53-6.7), αCD11b (M1/70), αCD11c (N418), αCD19 (eBio1D3), αCD25 (PC61.5), αCD45 (30-F11), αFoxP3 (FJK-16S), αNK1.1 (PK-136), αNkp46 (29A1.4), αLy6C (HK1.4), αLy6G (1A8), αCCR2 (R&D 475301), αγδ TCR (eBioGL3), αB220 (RA3-6B2). For intracellular αFoxP3 staining, cells were fixed after surface staining for 45 min and permeabilized for 30 min using the eBioscience FoxP3 staining kit. All flow cytometry data were acquired on a FACS Canto™ II (BD Bioscience) device and analyzed with the FlowJo software (v. 7.6.1, Tree Star Inc).

Blood samples of marmoset monkeys were analyzed by flow cytometry using the following directly labelled antibodies specific for: CD20 (B-Ly1, DakoCytomation), CD3 (SP34, BD Bioscience), CD14 (M5E2), CD11b (D12). For indirect staining, marmoset or human EDTA blood was first stained with the mouse-anti-human CCR2 antibody DOC-2 (5 µg/ml) followed by PE-labelled rabbit anti-mouse F(ab)2 (DakoCytomation), or with the humanized or marmoset chimeric DOC-2 Fr-2 followed by FITC-labelled goat anti human Fcγ specific F(ab’)2 (Jackson Immunoresearch).

### Blood–brain barrier permeability

Th/+ mice, healthy or previously immunized with rMOG_1–125_, were stereotactically injected with IFNγ and TNFα as described above and perfused 24 h afterwards. One hour before perfusion, the mice received i.v. injections of 100 µg FITC-albumin/g body weight (albumin–fluorescein isothiocyanate conjugate, Sigma, A9771).

### Histology and immunohistochemistry

*Mice* Mice were perfused transcardially at different time points: 24 h, day 5, day 10, day 20 and day 40 after cytokine injection with cold PBS followed by 4% paraformaldehyde (PFA). Brains and spinal cords were post-fixed for 2 days and then kept in PBS until dissected. Spinal cord and brain sections containing the injection site, identified by the trace of Monastral blue, were paraffin-embedded. Sections between 3 µm were cut and processed for immunohistochemistry (IHC) according to standard protocols. Cortical demyelination was evaluated on sections stained with a rabbit anti-myelin basic protein (MBP) antibody (Dako, A0623, 1:1000) using a Tyramide Signal Amplification kit (TSA™ Kit 21, Invitrogen). To characterize inflammatory infiltrates we stained with antibodies for T cells (rabbit anti-CD3, DCS, C1597C01, 1:100) and for microglia/macrophages (rat anti-Mac-3, BD Pharmingen™, Clone M3/84, 553322, 1:200).

To identify mature oligodendrocytes and oligodendrocyte precursor cells (OPC) in the cortical demyelinated areas, double-IHC was performed using a rabbit anti-TPPP/p25 antibody against tubulin polymerization-promoting protein (Abcam, 92305, 1:500) or a rabbit anti-Olig2 antibody (IBL, 18953, 1:300) respectively, on sections previously stained with rat anti-MOG antibody (purified in our laboratory). Double-IHC was also performed for myelin (anti-MOG) and amyloid precursor protein (APP, Chemicon, MAB348, 1:2000) as a marker of axonal damage or NeuN (Millipore, MAB377, 1:200) for neurons. Polymorphonuclear cells were detected by enzyme histochemistry using a chloroacetate esterase assay (Naphtol AS-D Chloroacetate esterase kit; Sigma-Aldrich).

NK cells infiltrating the cortex of stereotactically injected Th/+ mice were detected in cryosections using the primary goat anti-mouse antibody NKp46/NCR1 (R&D Systems, AF2225, 1:50). Briefly, mice where perfused with cold PBS. The brain was sectioned, mounted in OCT medium (Tissue-Tek; Sakura Finetek USA) and frozen in isopentane (Sigma-Aldrich) pre-cooled in liquid nitrogen. Serial sections 6 μm thick were cut in a cryostat at −20 °C and fixed in ethanol for 1 h before applying the NKp46 antibody. Antibody binding was visualized using DAB. To distinguish NK from NKT cells, we performed double-immunofluorescence on cryosections from stereotactically injected Th/+ mice using the goat anti-NKp46 antibody described above and a rabbit anti-CD3 antibody (Dako, A0452, 1:50). For visualizing NKp46, a donkey anti-goat biotinylated secondary antibody (GE Healthcare Ltd, RPN1025, 1:200) was added for 1 h at room temperature in 10% FCS/PBS, followed by 30 min incubation with Cy™3-conjugated streptavidin (Jackson ImmunoResearch Lab, 016160184, 1:100). The AlexaFluor^®^ 488 goat anti-rabbit antibody (Life Technologies, A11034, 1:200) was used to visualize the CD3 signal. Nuclei were counterstained with 4′,6-diamino-2-phenylindole (DAPI). Sections were examined using a fluorescence microscope (Olympus BX51, Germany).

Spinal cord sections from Th/+ CCR2^+/+^ and Th/+ CCR2^−/−^ were stained with Luxol fast blue (LFB)/periodic acid-Schiff (PAS) and evaluated for the extent of demyelination. Finally, paraffin-embedded brain sections from Th/+ mice injected with FITC-albumin were immunolabelled with a rabbit anti-FITC antibody conjugated to horseradish peroxidase (Dako, P5100, 1:50) to measure the area of FITC extravasation.

*Marmoset monkeys* Before transcardial perfusion with PBS and ice-cold 4% PFA, the animals received a lethal dose of ketamine/pentobarbital. Heads and spinal cords were post-fixed in 4% PFA, dissected and embedded in paraffin after 24 h. For the assessment of cortical demyelination and the identification of T cells in brain sections, we used the same primary antibodies as for mice (anti-MBP, enhanced with tyramide, and anti-CD3). For the detection of macrophages/monocytes the antibody MAC387 (mouse anti-L1 antibody MAC387, GeneTex, GTX6577, 1:150) was used. Spinal cord sections were in addition stained with LFB/PAS.

### Morphometric analysis

The quantification of cortical demyelination was performed on MBP-immunostained sections in mouse brains at the aforementioned time points. Images were acquired using a 40× magnification of a light microscope (Olympus BX51, Germany) equipped with a digital camera (Software CellSens Dimension v.1.7.1, DP71, Olympus, Germany). To measure cortical demyelination in the marmosets, MBP-immunolabelled sections were scanned using the Keyence Bio REVO BZ-X710 microscope (Keyence, Japan). Compound images of the entire coronal brain section analyzed were generated by combining single images using the BZ-II analyzer software (Keyence, Japan). ImageJ (v. 1.46r, NIH, USA) was used to measure the subpial demyelinated area and the extent of perivascular cortical demyelination in the digital composite images. In mice, subpial demyelination was quantified in the ipsilateral (injected) brain hemisphere, whereas perivascular demyelination was measured in both the ipsi- and contralateral cortex. Subpial demyelination (%) and the area of perivascular demyelination (mm^2^) are reported. Subpial demyelination was calculated as follows:

$${\text{Subpial demyelination}}\;(\% ) = \frac{{({\text{subpial demyelinated area}})}}{{({\text{total area of cortex}})}} \times 100.$$

Since cortical demyelination in mice was most extensive at day 5 post stereotactic injection, we selected this time point (if not stated otherwise) for quantitative analysis. Cell densities were determined using a 10 × 10 ocular morphometric grid (at 400× magnification) (Olympus, Japan), and the results were expressed as cells/mm^2^. The densities of p25+, Olig2+ and NeuN+ cells were assessed in the ipsilateral cortical layers I and II/III of C57BL6/J mice and in the subpial demyelinated areas across the same layers in Th/+ mice. The analysis of APP^+^ axons was restricted to the perivascular demyelinated areas in both hemispheres. For assessment of blood–brain barrier permeability the area of FITC-albumin extravasation was measured using ImageJ. To this end, a color deconvolution plugin for ImageJ was used [45], and the threshold established for quantifying the intensity of the brown (DAB) channel was set at (0; 150). For the analysis of macrophages/activated microglia in mice, the number of intracortical Mac-3+-perivascular cuffings was counted in both hemispheres. For the purpose of quantification, infiltrates restricted to the Virchow–Robin space, without evidence for cellular transmigration into the cortical parenchyma, were not considered *bona fide* perivascular infiltrates. The area of spinal cord demyelination in mice (Th/+ CCR2^+/+^, Th/+ CCR2^−/−^) and marmosets was measured on LFB/PAS stained sections using ImageJ. Digital images were acquired from at least eight transversal spinal cord sections from each animal, the demyelinated areas were measured and reported as % of total white matter. The densities of KiM1P+ macrophages/activated microglia and T cells in biopsies from early MS cases were determined using an ocular morphometric grid (Olympus, Japan). Immunostained cells were counted in regions with subpial cortical demyelination, as well as in normal appearing cortical regions (NAGM). At least five separate visual fields were quantified for each cortical region within each sample. Densities were reported as cells/mm^2^. The investigators (NLD, CS) were blinded to the experimental groups.

### qRT-PCR

Th/+ CCR2^+/+^ or Th/+ CCR2^−/−^ mice were perfused with PBS 2 days after stereotactic cytokine injection. Brains were dissected and the ipsilateral cortex near the injection site was separated from the white matter and conserved in QIAzol Lysis Reagent (Qiagen). RNA was isolated from the tissue using the RNeasy Micro Kit (Qiagen, Germany) and subsequently transcribed into cDNA using the High Capacity RNA-to-cDNA™ Kit (Life Technologies). From the cDNA, 12.5 ng were used per PCR reaction. Quantitative PCR was performed on the *iQ5*™ Real Time PCR Detection System (BIO-Rad) using the qPCR Core Kit obtained from Eurogentec. The following FAM labeled primers/probes (TaqMan^®^ Gene Expression Assays) were selected to be intron spanning from Life Technologies: Gapdh (Mm99999915 g1), TNF-α (Mm00443258_m1), NOS2/iNOS (Mm00440502_m1) and CCL2 (Mm00441242_m1). The expression levels of TNF-α, NOS2/iNOS and CCL2 are indicated as the percentage of the housekeeping gene Gapdh.

### Statistics

Normality tests (D’Agostino and Pearson omnibus normality test) were applied to all data sets. If the samples followed a normal distribution, the unpaired *t* test or a paired *t* test were applied. In the absence of normality, the non-parametric Mann–Whitney *U* test or the Wilcoxon signed-rank test were used. One-way-ANOVA followed by Dunn’s Multiple Comparison post hoc test was used for comparisons between three or more groups. *p* < 0.05 was considered to be significant. Unless otherwise specified, all results are shown as mean ± SD.

### Role of the funding source

The sponsors of this study had no role in study design, data collection, data analysis, data interpretation and writing of the manuscript. The corresponding authors had full access to all the data in the study and had final responsibility for the decision to submit the manuscript.

## Results

In our cohort of 740 biopsied patients with inflammatory white matter demyelination compatible with MS, 26 patients harbored subpial cortical demyelinated lesions, which extended from the subpial area to layers V–VI, reflecting 19% of the patients where cortex was available for microscopic analysis (Supplemental Fig. 1).

**Fig. 1:**
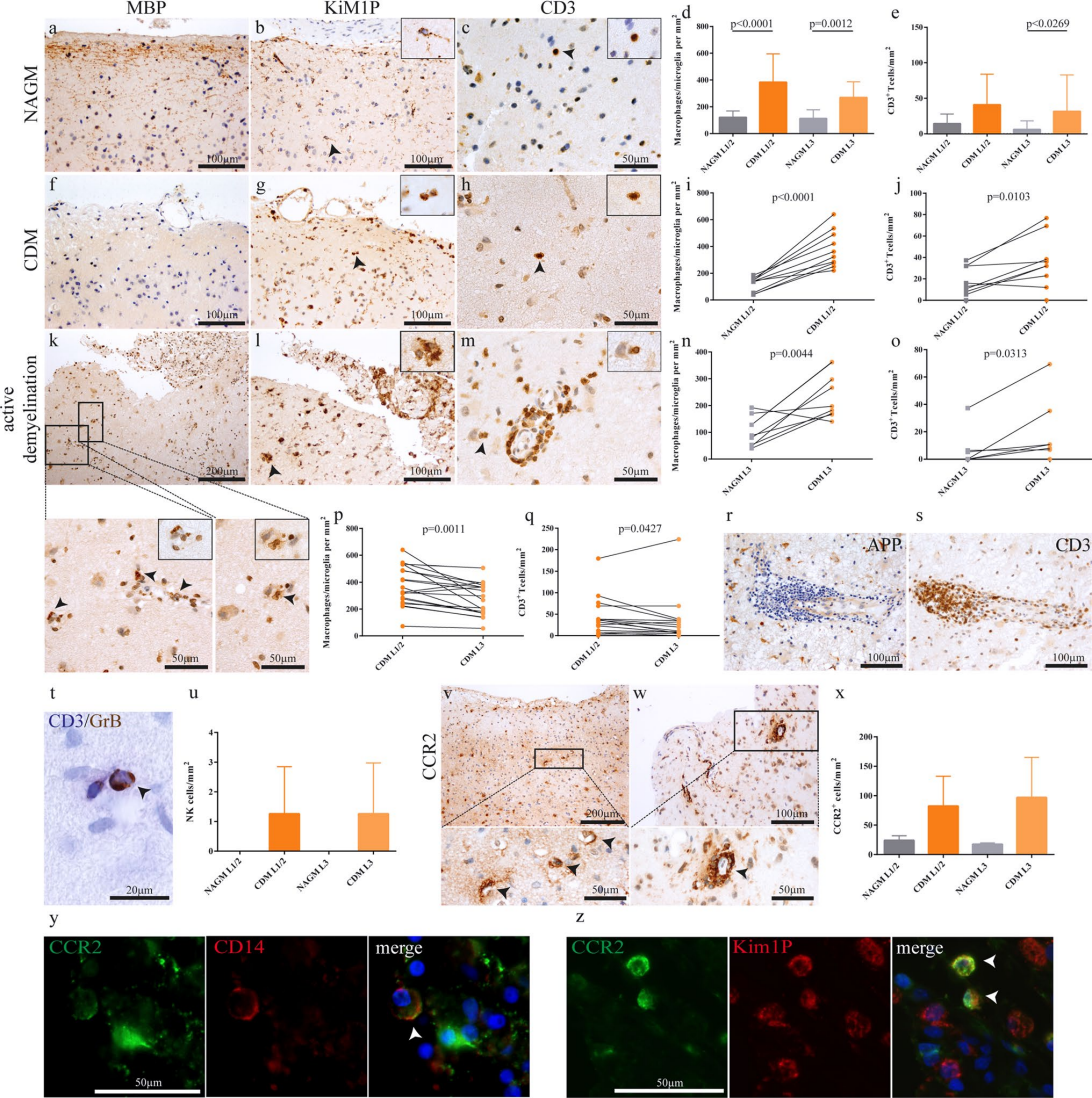
Increased adaptive and innate immune cell infiltration in demyelinated as compared to normal appearing cortical gray matter. In comparison with regularly myelinated normal appearing cortical *gray matter* (**a**–**c**), demyelinated cortex obtained at biopsy (**f**–**h**) shows higher densities of KiM1P+ macrophages/activated microglia (**d**, **g**, **i**, **n**) and T cells (**e**, **h**, **j**, **o**). An example of a cortical lesion with ongoing demyelination as evidenced by MBP-laden phagocytes (*arrows*, **k**–**m)**. In addition to foamy macrophages (**k**, **l**), T cells are found perivascularly, as well as in the parenchyma (**m**). A gradient of macrophage/microglia (**p**) and T cell densities (**q**) is apparent with higher densities in the superficial cortical layers 1/2 compared to layer 3. Multilayered perivascular T cell cuffs in the cortex, identified by the presence of APP^+^ neurons, were only seen in rare cases (**r**, **s**). The patient shown here presented with neuropsychiatric symptoms (patient no. 25). GrB^+^/CD3^−^ natural killer (NK) cells were observed perivascularly and only in demyelinated, but not normal appearing cortex (**t**, **u**, *arrow*). CCR2^+^ monocytes were mainly observed perivascularly, with some cells scattered in the parenchyma (**v**, **w**, **x**). CCR2/CD14 double-positive cells were observed perivascularly in patients with ongoing demyelination, indicating active myeloid cell recruitment into the cortex (**y**, *arrows*). Fluorescent double-immunohistochemistry of CCR2 and the pan-macrophage marker KiM1P identifies CCR2/KiM1P double-positive cells (**z)**. All data are presented as mean ± SD. **d**, **e** One-way ANOVA followed by Dunn's Multiple Comparison post hoc test; **i**, **j**, **n** paired *t* test; **o** Wilcoxon signed-rank test; **p**, **q** paired *t* test and Wilcoxon matched pairs signed-rank test. All immunohistochemistries were performed with diaminobenzidine (DAB; *brown color*), except for CD3 (*Fast Blue*, in double labelling with GrB). Cell nuclei are counterstained with hematoxylin. *APP* amyloid precursor protein, *NAGM* normal appearing cortical gray matter, *CDM* cortical demyelination, *GrB* granzyme B, *L1/2* cortical layer 1 and 2, *L3* cortical layer 3

We found 7/26 cortical demyelinated lesions with evidence for ongoing demyelination as indicated by the presence of MBP-laden phagocytes in all clinical MS subtypes (2 CIS, 4 RRMS, 1 SPMS). Foamy KiM1P^+^ macrophages/activated microglia were conspicuous in those cases (active and demyelinating according to Kuhlmann et al., Fig. 1l). The remaining 19 cortical demyelinated lesions were staged active and post-demyelinating [27]. The numbers of KiM1P+ macrophages/activated microglia observed in cortical demyelinated lesions significantly exceeded those found in the non-demyelinated, normal appearing cortex in both upper (L1/2) and lower (L3) cortical layers (Fig. 1d, i, n). Within the demyelinated cortex, densities of KiM1P^+^ cells were significantly higher in the immediate subpial cortical layer 1/2 as opposed to layer 3 (Fig. 1p). Macrophages in MS can be derived from either microglia cells or invading monocytes, and in white matter demyelination, monocyte-derived macrophages most likely predominate. We, therefore, performed CD14/CCR2 immunofluorescent double-labelling studies in eight cases (four with active cortical demyelination), in which tissue was available, to determine monocyte recruitment into the demyelinated cortex (Fig. 1y). Three main populations could be distinguished: CD14^−^ CCR2^+^ cells often with rounded, amoeboid morphology were found perivascularly, as well as scattered in the cortical parenchyma. CD14^−^ CCR2^+^ cells were Iba-1^+^, but most of the CCR2^+^ cells (>95%) were negative for the microglia specific markers TMEM119 and P2Y_12_ and the pan T cell marker CD3. A proportion of CCR2^+^ cells showed foam cell morphology and co-expressed KiM1P. CD14^+^ CCR2^+^ cells were more restricted and mainly identified perivascularly in 4/8 cases, and a predominantly perivascular distribution was also observed for CD14^+^ CCR2^−^ cells. Numbers of CCR2^+^ cells were low in the normal appearing cortex and increased in demyelinated areas (Fig. 1v–z, Supplemental Fig. 2).

Infiltration with CD3^+^ T cells was significantly higher in demyelinated compared to normal-appearing cortical gray matter in both cortical layers 1/2 and 3 (Fig. 1e, j, o), and within the demyelinated cortex, CD3^+^ T cell numbers were higher in cortical layer L1/2 than in cortical layer L3 (Fig. 1q). In our cohort, few patients with substantial perivascular lymphocyte infiltration could be identified (Fig. 1s). CD4 and CD8 T cell subsets were present in similar frequencies (Supplemental Fig. 3). Few GrB^+^ CD3^−^ NK cells were predominantly detected perivascularly and rarely in the cortical parenchyma, but not in the non-demyelinated cortex (Fig. 1t, u).

**Fig. 2:**
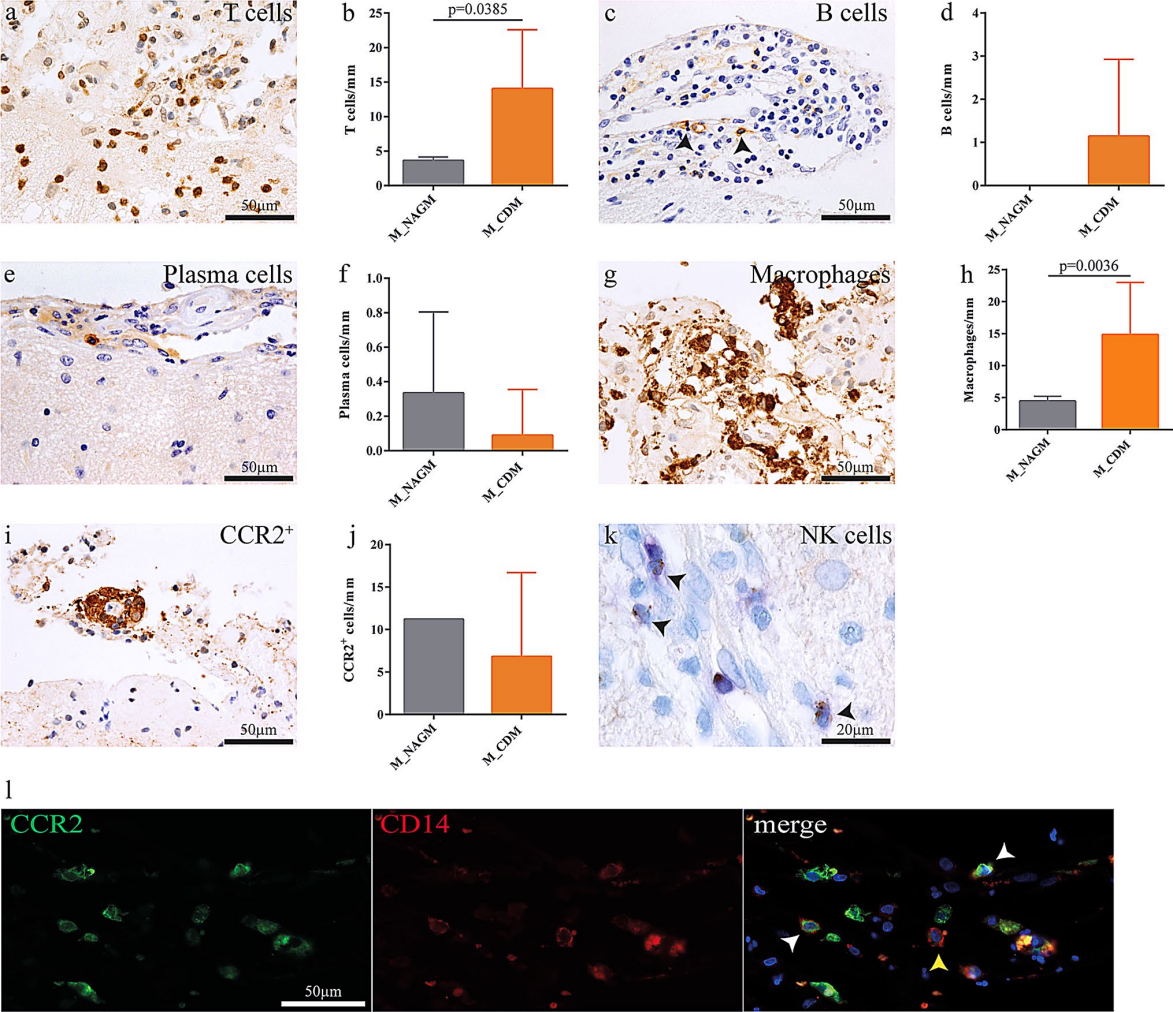
The intensity of meningeal inflammation associates with cortical demyelination. Meningeal inflammation, mostly arranged in a perivascular fashion, was found adjacent to demyelinated and non-demyelinated cortical areas. T lymphocyte (**a**, **b**) and macrophage (**g**, **h**) infiltration was significantly higher in areas overlying demyelinated cortex. Also, macrophages and T cells were more abundant than B cells (**c**, **d**) and plasma cells (**e**, **f**). NK cells were exceedingly rare (**k**). CCR2^+^ monocytes were found perivascularly in the leptomeninges overlying both demyelinated and normal-appearing cortical gray matter (**i**, **j**). Meningeal CD14^+^ CCR2^+^ and CD14^+^ CCR2^−^ monocytes overlying an actively demyelinating cortical lesion in a patient with monocyte invasion into the subpial cortex (**l**). Data are presented as mean ± SD; Mann–Whitney test

Leptomeninges overlying subpial cortical demyelination were present in 13 of our 26 patients and displayed higher numbers of macrophages (Fig. 2g, h), T cells (Fig. 2a, b) and B cells (Fig. 2c, d) compared to those localized adjacent to non-demyelinated cortical tissue (Fig. 2). Also, CCR2^+^ monocytes were found, mainly perivascularly, in the leptomeninges overlying demyelinated cortex (Fig. 2i). However, tissue of only one patient comprising leptomeninges overlying normal-appearing cortex was available for CCR2 IHC for comparison (Fig. 2j). Leptomeningeal plasma cells were found adjacent to demyelinated and non-demyelinated cortex (Fig. 2e, f), and NK cells were exceedingly rare (Fig. 2k). In a highly inflammatory cortically demyelinated MS patient CD14^+^ CCR2^+^ and CD14^+^ CCR2^−^ monocytes were found to directly infiltrate the subpial brain parenchyma (Fig. 2l).

In summary, our study in a cohort of early and in part actively demyelinating cortical lesions demonstrates both adaptive and innate inflammatory cells in the cortex as well as the overlying meninges and presents evidence for early invasion of CD14^+^ CCR2^+^ and CD14^+^ CCR2^−^ monocytes. Furthermore, a gradient of inflammatory cell infiltration was observed, with higher densities in the subpial cortex.

### Cortical demyelination requires a conformation-specific antibody response against myelin

**Fig. 3:**
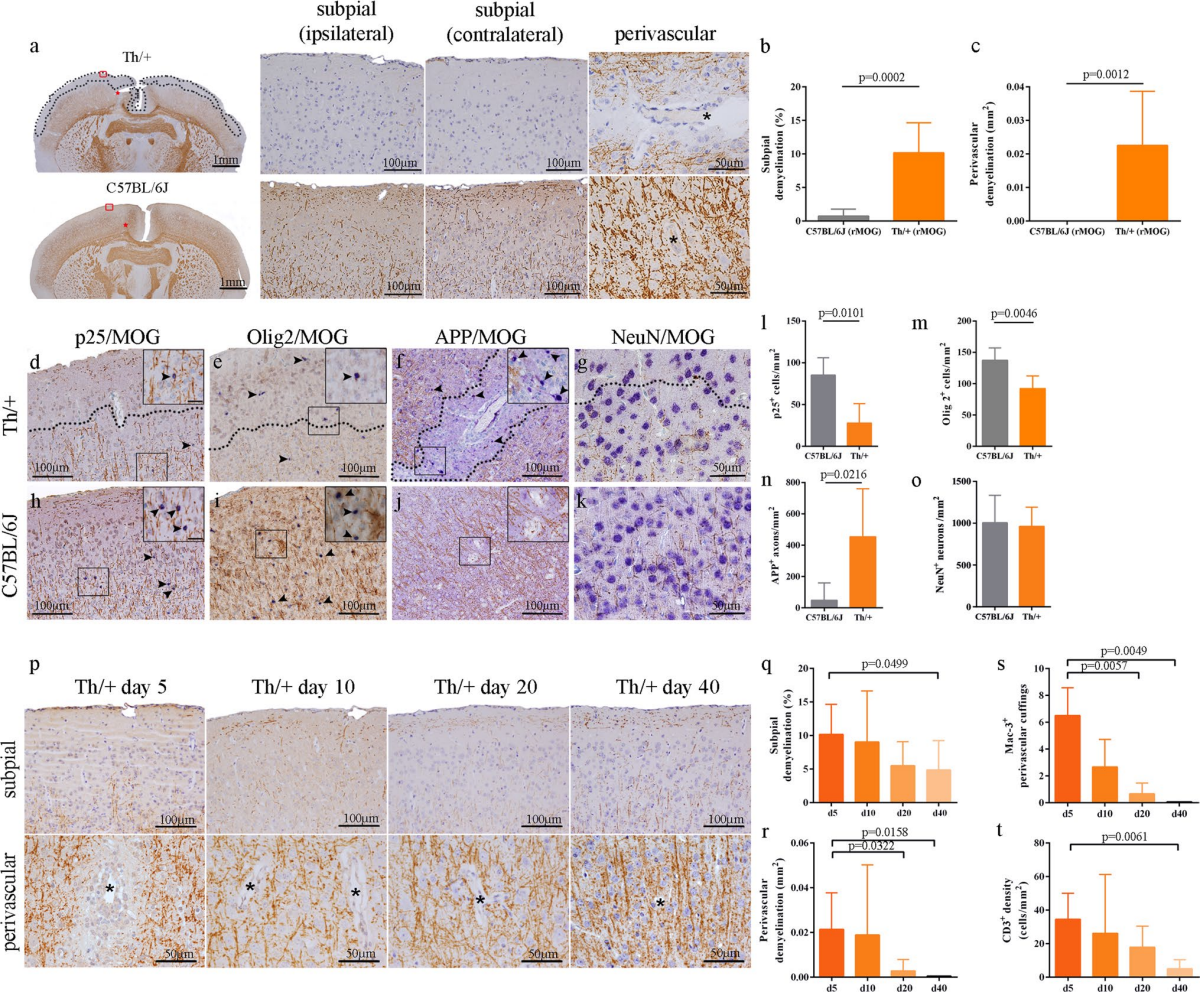
Cortical demyelination in Th/+ mice with demyelinating antibodies. **a** Subpial and perivascular cortical demyelination as assessed by immunohistochemistry (IHC) for MBP (*brown color*) on day 5 after stereotactic cytokine injection in Th/+ mice (*top*) and C57BL/6J mice (*bottom*). **b, c** Subpial cortical demyelination was quantified on MBP-immunostained brain sections as percentage of total cortical area of the injected hemisphere. The extent of perivascular cortical demyelination is given in mm^2^ (C57BL/6: *n* = 5; Th/+: *n* = 13). Data are from three independent experiments and presented as mean ± SD; Mann–Whitney test. Representative IHC of mature oligodendrocytes (**d**, **h**), oligodendroglial cells including OPC (**e**, **i**), axons (**f**, **j**) and neurons (**g**, **k**) in cortical demyelinated areas of Th/+ (*top*) and corresponding non-demyelinated cortex in C57BL/6J mice (*bottom*). Cell markers are depicted in *blue* (*Fast Blue*), myelin oligodendrocyte glycoprotein (MOG) in *brown* (DAB). Exemplary APP^+^ axons as well as p25^+^ and Olig2^+^ cells are indicated by *arrowheads* and shown in greater detail (*insets*). *Dotted lines* in the images mark the *border* between demyelinated and non-demyelinated cortical areas. *Scale bars*: *insets* 20 µm. Densities of p25^+^ oligodendrocytes (**l**), Olig2^+^ cells (**m**), APP^+^ damaged axons (**n**) and NeuN^+^ neurons (**o**) were determined in subpial cortical demyelination in Th/+ mice and corresponding cortical layers in C57BL/6J mice. A total of 7 Th/+ and 5 C57BL/6J mice were analyzed in two independent experiments and are presented as mean ± SD; Mann–Whitney test. **p**–**t** Resolution of inflammation and lesion repair of cortical demyelination in Th/+ mice. The extent of subpial and perivascular cortical demyelinated lesions was assessed by MBP IHC on days 5, 10, 20 and 40 post cytokine injection (**p**). *Asterisks* mark parenchymal vessels. (**q**, **r**) Quantitative analysis of subpial cortical demyelination and perivascular cortical demyelination (d5: *n* = 13, three independent experiments; days 10, 20, 40: *n* = 6 each, two independent experiments). Data are presented as mean ± SD; one-way ANOVA followed by Dunn's Multiple Comparison post hoc analysis. Quantification of Mac-3 (macrophages, **s**) and CD3 (T cells, **t**) IHC on days 5, 10, 20 and 40 post stereotactic cortical cytokine injections. Representative data of two independent experiments for each time point presented as mean ± SD. Mac-3 (*n* = 6, for each day), T cells (*n* = 6, d5 and d20; *n* = 5, d10 and d40); one-way ANOVA followed by Dunn's Multiple Comparison post hoc analysis

In a next step, we strived to model cortical demyelination in the mouse to obtain an easily manipulable system in which the relative contribution of adaptive and innate immune cells as well as humoral components for cortical demyelination can be assessed. Cortical demyelination is not a regular feature of experimental autoimmune encephalomyelitis (EAE) in MOG_35–55_-peptide- or rMOG_1–125_-immunized C57BL/6 mice. To mimic the pro-inflammatory cytokine milieu found in the CSF of patients with cortical demyelination, we stereotactically injected TNFα and IFNγ into the right motor cortex of MOG_35–55_-peptide immunized 2D2 mice (TCR transgenic for MOG), rMOG_1–125_-immunized C57BL/6 mice or rMOG_1–125_-immunized Th/+ mice (BCR transgenic for MOG) at the 2nd day of clinical EAE. Subpial and perivascular cortical demyelination were only observed in Th/+ mice (Fig. 3a), which carry the Ig heavy chain knockin of 8-18C5, an established demyelinating MOG-specific antibody. Of note, Th/+ mice developed cortical demyelination subpially and intracortically in both the injected and non-injected hemispheres (Fig. 3a–c). Demyelination in the cortex was most pronounced on day 5 after injection and paralleled by a significant reduction in p25^+^ mature oligodendrocytes (Fig. 3d, h, l). In line with previous studies demonstrating axonal damage in cortical MS lesions, the density of damaged APP^+^ axons was significantly increased in perivascular cortical demyelination of Th/+ mice compared to corresponding cortical regions in C57BL/6J mice (Fig. 3f, j, n), while the density of neurons in the demyelinated cortex was not reduced (Fig. 3g, k, o). A significant decline in perivascular macrophage cuffings and T cell densities could be observed on days 20 and 40 after lesion induction (Fig. 3s, t), and lesions were significantly remyelinated on day 20 perivascularly and on day 40 subpially (Fig. 3p–r). Concordant with the maximum of demyelination observed, cortically demyelinated Th/+ mice were impaired in the motor skill sequence (MOSS) test between days 4 and 6 after lesion induction (Supplemental Fig. 4).

### The classical pathway of complement activation is dispensible for cortical demyelination in Th/+ mice

**Fig. 4:**
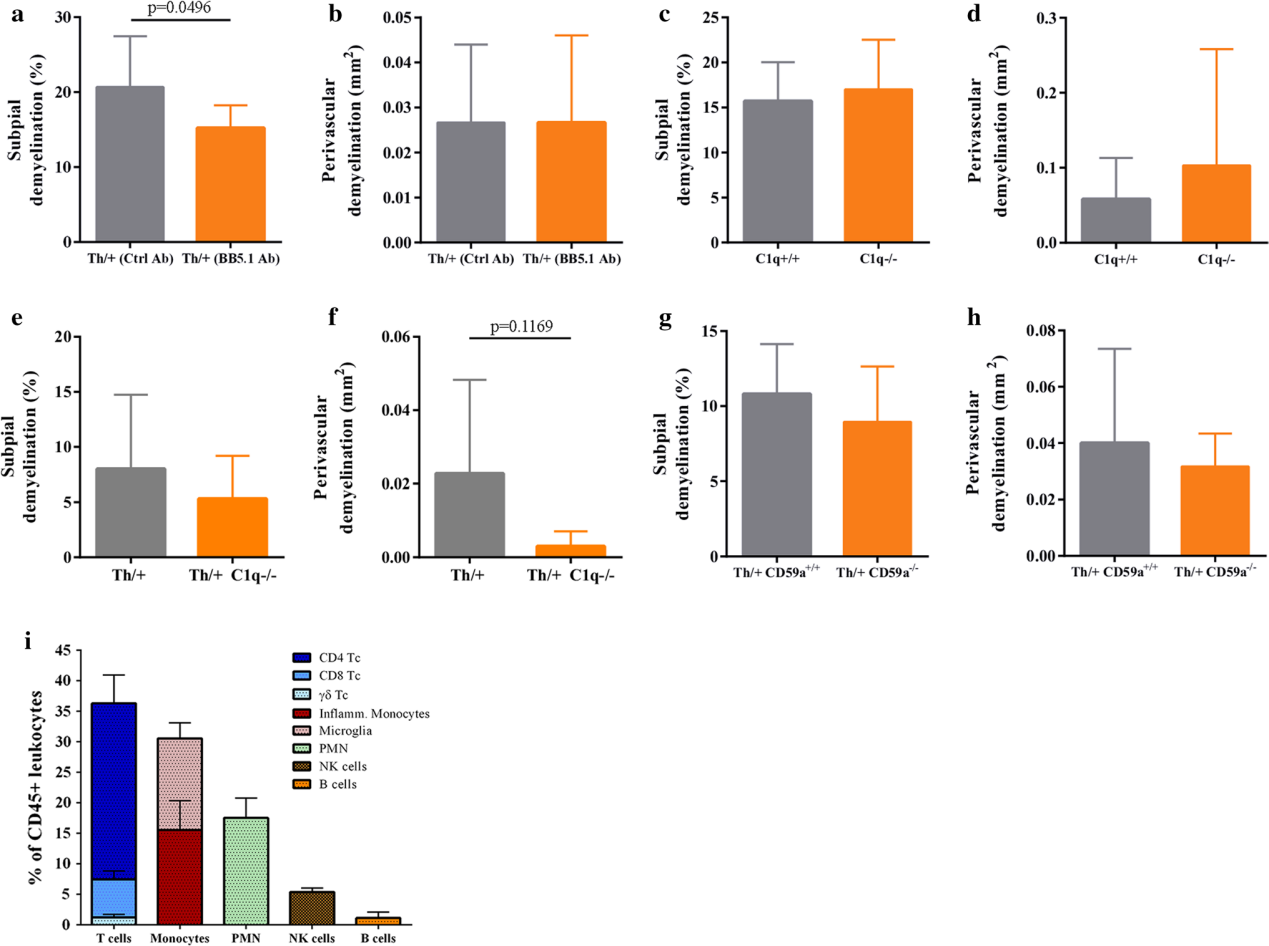
The classical complement pathway does not substantially contribute to cortical demyelination. **a**, **b** Quantitative assessment of subpial and perivascular cortical demyelination in MBP-immunostained brain sections of cytokine injected Th/+ mice, which received the C5 convertase blocking antibody BB5.1 or an isotype control antibody (*n* = 9 animals/group, analyzed in two independent experiments; unpaired *t* test). **c**, **d** Quantitative assessment of subpial and perivascular cortical demyelination in MBP-immunostained brain sections of C1q^−/−^ or C57BL/6 (C1q^+/+^) mice, which received 1.5 mg of the MOG-specific, complement-fixing antibody Z2 i.v. prior to stereotactic cytokine injections. **e**, **f** Quantitative assessment of subpial and perivascular cortical demyelination in MBP-immunostained brain sections of Th/+ C1q^−/−^ (*n* = 6) and Th/+ C1q^+/+^ (*n* = 9) mice; representative data of two independent experiments. **g**, **h** Quantitative assessment of subpial and perivascular cortical demyelination in MBP-immunostained brain sections of Th/+ CD59a^−/−^ and Th/+ CD59a^+/+^ mice; *n* = 9/group, representative data of two independent experiments. **i** Leukocyte subsets in cortical demyelination. Leukocytes isolated from the demyelinated cortex of Th/+ mice (*n* = 3) were analyzed by multicolor flow cytometry on day 2 post injection. All data are presented as mean ± SD

MOG-specific antibodies targeting conformational epitopes are a prerequisite for cortical demyelination in our animal model. They might bind to their cognate antigen on the cell surface of oligodendrocytes and activate C1q, the first component of the classical complement pathway that leads to target cell killing by complement-dependent cytotoxicity (CDC) via membrane attack complexes. In addition, elimination of antibody-opsonized cells might be mediated by NK cells, macrophages, monocytes and neutrophils, which are activated via specific IgG receptors sensing surface bound IgG antibodies. To assess the relative importance of humoral and cellular effector mechanisms for the pathogenesis of myelin damage in subpial and perivascular cortical demyelination, we first strived to detect the membrane attack complex in cortical demyelinated lesions in human MS biopsies. Although we detected subpial IgG leakage in patients with ongoing cortical demyelination (Supplemental Fig. 5 a, c), no evidence of terminal complement activation was found (not depicted). Next, we prevented the generation of the membrane attack complex (MAC) in our animal model by a specific antibody directed against C5 (BB5.1, the mouse analogue of eculizumab) thereby inhibiting its cleavage [51]. Inhibiting terminal complement activation reduced subpial cortical demyelination, whereas the extent of perivascular cortical demyelination was not modulated (Fig. 4a, b). If we compared cortical demyelination in C1q-deficient mice and controls with EAE which received the potent complement-activating and MOG-specific antibody Z2 i.v. prior to stereotactic cytokine injections, the extent of cortical demyelination in C1q-deficient animals was equivalent to controls (Fig. 4c, d). To gain further insight whether CDC is relevant for cortical demyelination in Th/+ mice, we established Th/+ C1q^−/−^ and Th/+ CD59a^−/−^ mice, the latter being deficient for a major membrane inhibitor of terminal complement activation in oligodendrocytes. Th/+ C1q^−/−^ and Th/+ C1q^+/+^ mice did neither differ in MOG-specific antibody titres (not depicted) nor in the extent of cortical demyelination (Fig. 4e, f). Furthermore, the extent of cortical demyelination was similar in Th/+ CD59a^−/−^ and Th/+ CD59a^+/+^ mice (Fig. 4g, h). In summary, we provide evidence that CDC activated via the classical complement pathway unlikely contributes to cortical demyelination in Th/+ mice.

We further characterized the cortical inflammatory infiltrate by flow cytometry and identified both adaptive (T and B cells) and innate immune cells (monocytes, granulocytes (PMN) and natural killer (NK) cells) in the demyelinated cortex of Th/+ mice on day 3 after lesion induction (Fig. 4i). To gain insight into the relevance of individual immune cell populations for cortical demyelination, we selectively depleted individual immune cells by specific antibodies or used transgenic mice genetically deficient for adaptive immune cells, NK cells or inflammatory monocytes.

### Natural killer cells contribute to perivascular cortical demyelination

**Fig. 5:**
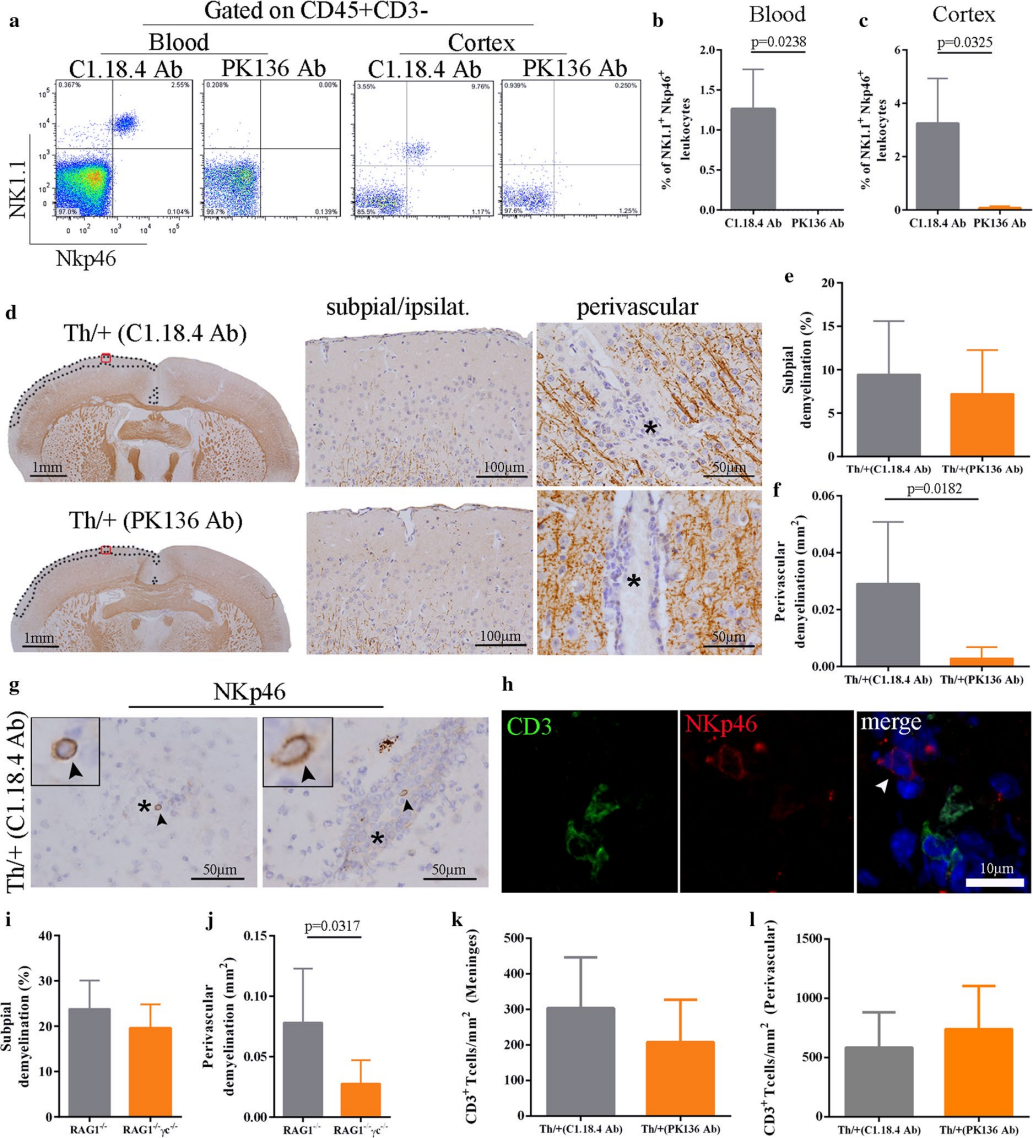
NK cells contribute to perivascular cortical demyelination. **a**–**c** Analysis of NK depletion efficiency in the blood (before stereotactic injection) and in the cortex (d5 after stereotactic injection) of Th/+ mice by multicolor flow cytometry. **b**, **c** Quantification of NK cells in the blood (**b**) and the cortex (**c**) of NK cell depleted (PK136 antibody, *n* = 4) and control mice (C1.18.4 antibody, *n* = 3). Data are given as mean ± SD and were analyzed in two independent experiments; Mann–Whitney test. **d** Subpial and perivascular cortical demyelination as assessed by IHC for MBP (*brown color*) on day 5 after stereotactic cytokine injection in NK cell depleted Th/+ mice (PK136 antibody, *bottom*) and controls (C.1.18.4 antibody, *top*). *Red squares* in the brain overviews mark the magnified areas in the subpial ipsilateral photographs. *Dotted lines* define the respective subpial demyelinated areas. Vessel lumina are marked by asterisks. **e**, **f** Quantitative analysis of subpial and perivascular cortical demyelination in MBP-immunostained brain sections of 7 animals per group. Data are presented as mean ± SD and were analyzed in two independent experiments; unpaired *t* test with Welch correction. **g** Representative IHC of perivascularly located NK cells (NKp46, *arrowheads*) in the cortex of Th/+ mice. *Asterisks* mark the vessel lumen. **h** Immunohistochemical double-labelling of NKp46 (*red*) and CD3 (*green*). A perivascular NK cell is marked with a *white arrowhead*. Nuclei were counterstained with DAPI (*blue signal*). **i**, **j** Quantitative analysis of subpial and perivascular cortical demyelination in 2D2 T cell and anti-MOG antibody (Z2) transferred RAG1^−/−^ (*n* = 5) and RAG1^−/−^ γc^−/−^ mice (*n* = 4). **k**, **l** Quantitative analysis of meningeal and perivascular CD3^+^ T cells in NK cell depleted Th/+ mice and controls. Data are presented as mean ± SD analyzed in three independent experiments; Mann–Whitney test

NK cells are the classical effectors of antibody-dependent cellular cytotoxicity (ADCC) against IgG-coated targets and were detected around vessels in both demyelinated cortical lesions of MS biopsies (Fig. 1t) and in cortically demyelinated Th/+ mice (Fig. 5g, h). When we efficiently depleted NK cells in the blood and cortex of Th/+ mice prior to stereotactic surgery (Fig. 5a–c), the extent of subpial cortical demyelination was unaffected by NK cell depletion, but perivascular cortical demyelination was significantly diminished (Fig. 5d–f). Of note, NK cell depletion did not affect the numbers of perivascular or meningeal CD3^+^ T cells (Fig. 5k, l). To further support the relevance of NK cells to perivascular cortical demyelination, we adoptively transferred activated MOG-specific 2D2 T cells into RAG1^−/−^ (no mature T and B cells) and RAG1^−/−^ γc^−/−^ (no mature T and B cells, no NK cells) mice. At disease onset, all animals received 1.5 mg of the demyelinating MOG-specific IgG2a antibody Z2 i.v. and underwent stereotactic cytokine injections into the motor cortex. Whereas RAG1^−/−^ and RAG1^−/−^ γc^−/−^ mice developed similar subpial cortical demyelination, perivascular cortical demyelination was significantly higher in RAG1^−/−^ compared to RAG1^−/−^ γc^−/−^ animals (Fig. 5i, j). Our experiments thus demonstrate that NK cells are relevant for perivascular cortical demyelination in the presence of a pathogenic antibody response, which is in line with their predominant perivascular localization in the inflamed cortex.

### Encephalitogenic T cells are dispensable for subpial type 3, but not perivascular type 2 cortical demyelination

**Fig. 6:**
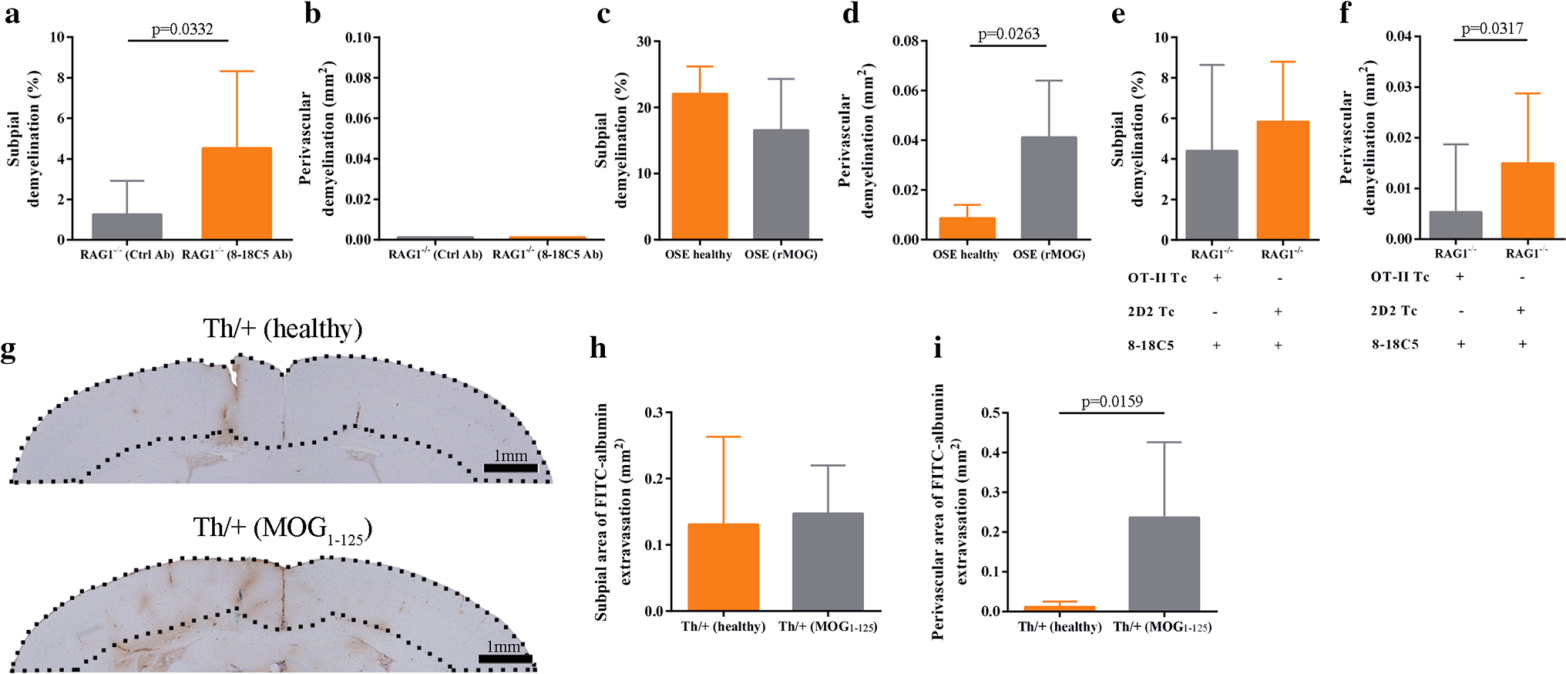
Encephalitogenic T cells are dispensable for subpial but not for perivascular demyelination. **a**, **b** Quantitative analysis of subpial and perivascular cortical demyelination in 8–18C5 (*n* = 8) and control antibody (*n* = 9) transferred RAG1^−/−^ mice analyzed in two independent experiments. Data are presented as mean ± SD; unpaired *t* test. **c**, **d** Quantitative analysis of subpial and perivascular cortical demyelination in healthy (*n* = 4) and diseased OSE mice (*n* = 8) analyzed in two independent experiments. Data are given as mean ± SD; Mann–Whitney test. **e**, **f** Quantitative analysis of subpial and perivascular cortical demyelination in 2D2 Tc/8-18C5 antibody transferred RAG1^−/−^ mice (*n* = 6) and OT-II Tc/8-18C5 antibody transferred RAG1^−/−^ mice (*n* = 12). Representative data of two independent experiments are presented as mean ± SD; Mann–Whitney test. **g** Assessment of FITC-albumin extravasation by IHC in healthy (*top*) and diseased Th/+ mice (*bottom*) 24 h after stereotactic cytokine injection into the motor cortex. *Dotted lines* mark the cortical area where FITC-albumin extravasation was quantified. **h**, **i** Quantification of FITC-albumin extravasation in healthy (*n* = 4) and diseased Th/+ mice (*n* = 5). Data are presented as mean ± SD; Mann–Whitney test

Clinical trials in MS have been interpreted in a way that the progressive disease phase might be less dependent on peripherally recruited adaptive immune cells. Since cortical demyelination is prominent in chronic MS, we scrutinized whether T cells are central to cortical lesion formation in our model. Also, we wanted to clarify, whether CNS-antigen specificity and T cell activation influence cortical demyelination. To this end, we combined T- and B cell receptor transgenic mice for MOG (OSE: TCR^MOG^ × IgG^MOG^) and transferred activated MOG-specific T cells (TCR^MOG^, named 2D2) or activated, ovalbumin-specific T cells (OT-II, which recognize an antigen not present in mice) into mice deficient for adaptive immune cells (RAG1^−/−^ mice). Surprisingly, we found that T and B cells were not immediately required for subpial cortical demyelination in the presence of demyelinating antibodies and stereotactically applied cytokines (Fig. 6a, b). However, activated CNS-antigen specific T cells were a prerequisite for perivascular cortical demyelination (Fig. 6c–f). These results were supported by experiments assessing the permeability of the cortical blood–brain barrier via the extravasation of FITC-albumin in healthy, non-immunized Th/+ mice versus diseased Th/+ mice harboring CNS-antigen specific, activated T cells (Fig. 6g). Perivascular cortical extravasation of FITC-albumin was significantly more pronounced in diseased than in healthy Th/+ mice, whereas subpial extravasation of serum proteins was substantial in both experimental paradigms 24 h after lesion induction (Fig. 6h, i). This indicates that intracortically applied cytokines are sufficient to increase the permeability of meningeal and subpial vessels readily while intracortical vessels require the combined action of cytokines and encephalitogenic T cells to become permeable to serum proteins, such as albumin and IgG. In line, IgG leakage was detected subpially in a single patient with ongoing, active cortical demyelination (Supplemental Fig. 5).

### CCR2^+^ monocytes are required for inflammatory cortical demyelination

**Fig. 7:**
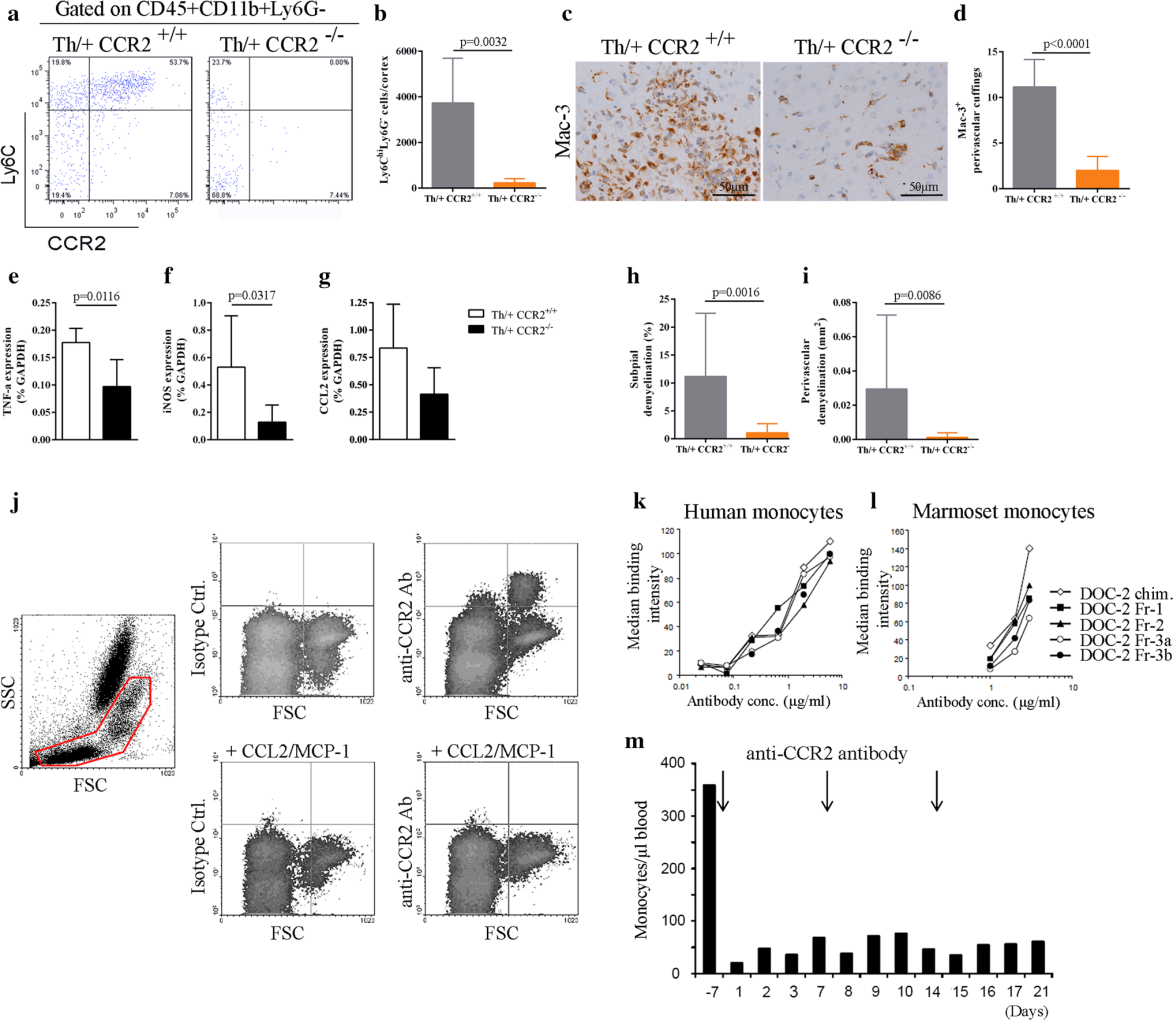
Inflammatory monocytes are indispensable for cortical demyelination in Th/+ mice. **a** Representative density plots of inflammatory monocytes (CD45^+^CD11b^+^Ly6C^hi^Ly6G^-^) isolated from the cortex of Th/+ CCR2^+/+^ and Th/+ CCR2^−/−^ mice on day 2 post stereotactic injection. **b** Cortical inflammatory monocytes were quantified by flow cytometry in seven animals/group analyzed in three independent experiments and presented as mean ± SD; unpaired *t* test with Welch correction. **c** Representative IHC for activated microglia/macrophages (Mac-3) in Th/+ CCR2^+/+^ mice (left) and Th/+ CCR2^−/−^ mice (*right*). **d** The number of Mac-3^+^ intracortical perivascular cuffings was assessed in Th/+ CCR2^+/+^ and Th/+ CCR2^−/−^ mice and presented as mean ± SD; *n* = 6 mice per group, analyzed in two independent experiments; unpaired *t* test. **e–g** Quantitative real-time PCR analysis of monocyte-related cytokines and chemokines in the cortex of Th/+ CCR2^+/+^ (*white bars*) and Th/+ CCR2^−/–^ mice (*black bars*) on day 2 after stereotactic injection. Results are normalized against GAPDH expression. One representative experiment is shown, *n* = 5 animals per group; Mann–Whitney test. **h**, **i** Quantification of subpial and perivascular cortical demyelination in Th/+ CCR2^+/+^ (*n* = 13) and Th/+ CCR2^−/−^ mice (*n* = 5). Data of three independent experiments are shown and expressed as mean ± SD. Mann–Whitney test. **j** Representative flow cytometry dot plots of marmoset blood. The monoclonal mouse anti-human CCR2 antibody DOC-2 (*upper panel right*), but not the isotype control antibody (*upper panel left*) binds to marmoset monocytes. Binding of DOC-2 antibodies to monocytes is lost, if CCR2 is internalized by pretreatment of the cells with CCL2 (1 µg/ml) for 30 min at 37 °C (*lower panel*), demonstrating target specificity. **k**, **l** Representative plot for titrated humanized DOC-2 antibodies (named DOC-2 Fr) demonstrating their preserved capability to bind to human (*left panel*) and marmoset monocytes (*right panel*) after humanization. **m** Monocyte concentrations in the blood of healthy marmosets treated weekly with 5 mg/kg i.v. of DOC-2 Fr-2

Quantitatively, macrophages are by far the dominant inflammatory cell population in MS and correlate with active demyelination. Recent evidence has been provided that blood-derived CCR2^rfp/+^ monocytes give rise to highly phagocytic and inflammatory macrophages, which initiate demyelination in EAE [53]. To assess the contribution of CCR2^+^ monocytes for cortical demyelination, Th/+ CCR2^−/−^ mice and Th/+ CCR2^+/+^ controls were immunized with rMOG_1–125_ and received stereotactic injections on day 2 after EAE onset. In line with previous reports in CCR2-deficient animals [17], Th/+ CCR2^−/−^ mice developed EAE at a comparable severity, albeit at later time points than Th/+ CCR2^+/+^ controls (Table S2). The recruitment of inflammatory monocytes into the cortex was significantly impaired in Th/+ CCR2^−/−^ mice compared to Th/+ CCR2^+/+^ controls on day 2 after stereotactic injection (Fig. 7a, b). Consistent with the flow cytometry data, Th/+ CCR2^−/−^ mice had significantly less intracortical Mac-3+ perivascular cuffings in both hemispheres than Th/+ CCR2^+/+^ mice (Fig. 7c, d) and reduced mRNA levels of monocyte-related genes such as TNFα and iNOS (Fig. 7e–g). Most importantly, both subpial cortical demyelination and perivascular cortical demyelination were significantly reduced in Th/+ CCR2^−/−^ compared to Th/+ CCR2^+/+^ mice (Fig. 7h, i).

### Depletion of CCR2^+^ monocytes reduces cortical demyelination in marmosets with EAE

**Fig. 8:**
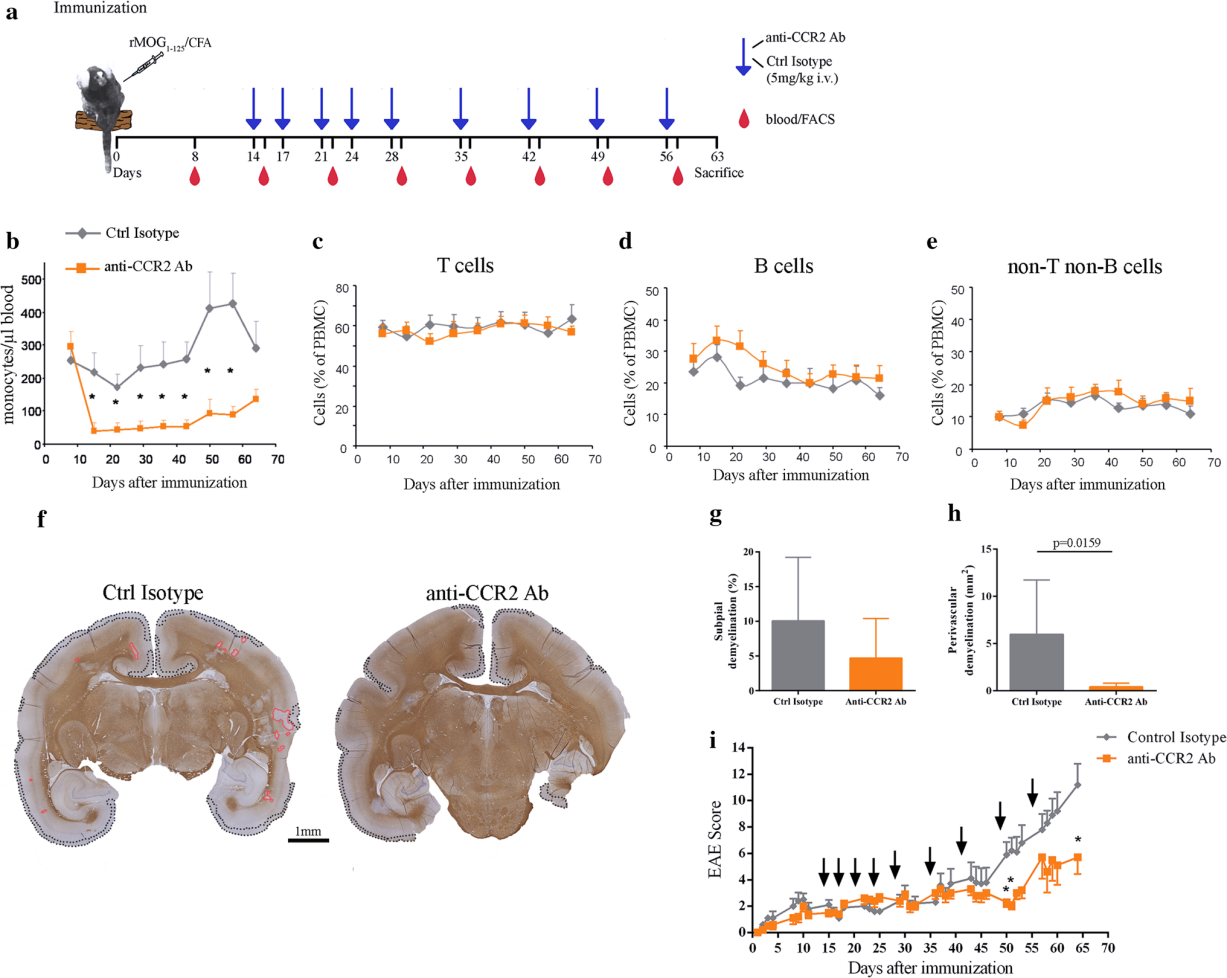
Depletion of CCR2+ monocytes in marmosets with EAE ameliorates cortical demyelination. **a** Schematic representation of the treatment protocol for marmoset monkeys. **b**–**e** Analysis of immune cell subpopulations in the blood of marmosets with EAE in response to injections of 5 mg/kg DOC-2 Fr2 (twice weekly d14–d28; once weekly d28-56). **f** Representative overview of coronal brain sections immunostained for MBP (*brown*). Subpial cortical demyelinated regions (*black dotted lines*) and perivascular cortical demyelinated areas (*red lines*) are highlighted. **g**, **h** The extent of subpial and perivascular cortical demyelination was measured on MBP-immunostained sections of DOC-2 Fr-2 (anti-CCR2, *n* = 5) and Ctrl-antibody (*n* = 5) injected marmosets; Mann–Whitney test. **i** Clinical disease severity of anti-CCR2 or isotype control treated marmosets (*n* = 5/group; *p < 0.05, one sided Mann–Whitney test)

The detection of CD14^+^ CCR2^+^ double-positive monocytes invading the cortex with ongoing, active demyelination (Figs. 1y, 2l) let us hypothesize that targeting CCR2 might represent a valid treatment strategy against cortical demyelination. We, therefore, developed a novel monoclonal mouse anti-human CCR2 antibody (DOC-2) cross-reacting with marmoset CCR2 (Fig. 7j–l). After humanization, DOC-2 antibodies (now named DOC-2 Fr) retained their capability to bind to human and marmoset monocytes (Fig. 7k, l), were well tolerated, and efficiently depleted monocytes in healthy marmosets at a dose of 5 mg/kg given weekly i.v. (Fig. 7m). Next, we specifically depleted CCR2^+^ monocytes (but not T and B cells) in marmosets with EAE using twice weekly i.v. injections of DOC-2 Fr-2 starting 2 weeks after immunization (Fig. 8a–e). Monocyte-depleted animals displayed less subpial type 3 cortical demyelination and significantly less perivascular type 2 cortical demyelination compared to isotype-treated controls (Fig. 8f–h), in line with an ameliorated clinical disease course (Fig. 8i). In summary, these observations provide evidence that inflammatory monocytes are crucial for the initiation of cortical demyelination and thus represent a specific and rational therapeutic target for the reduction of inflammatory cortical pathology.

## Discussion

**Fig. 9:**
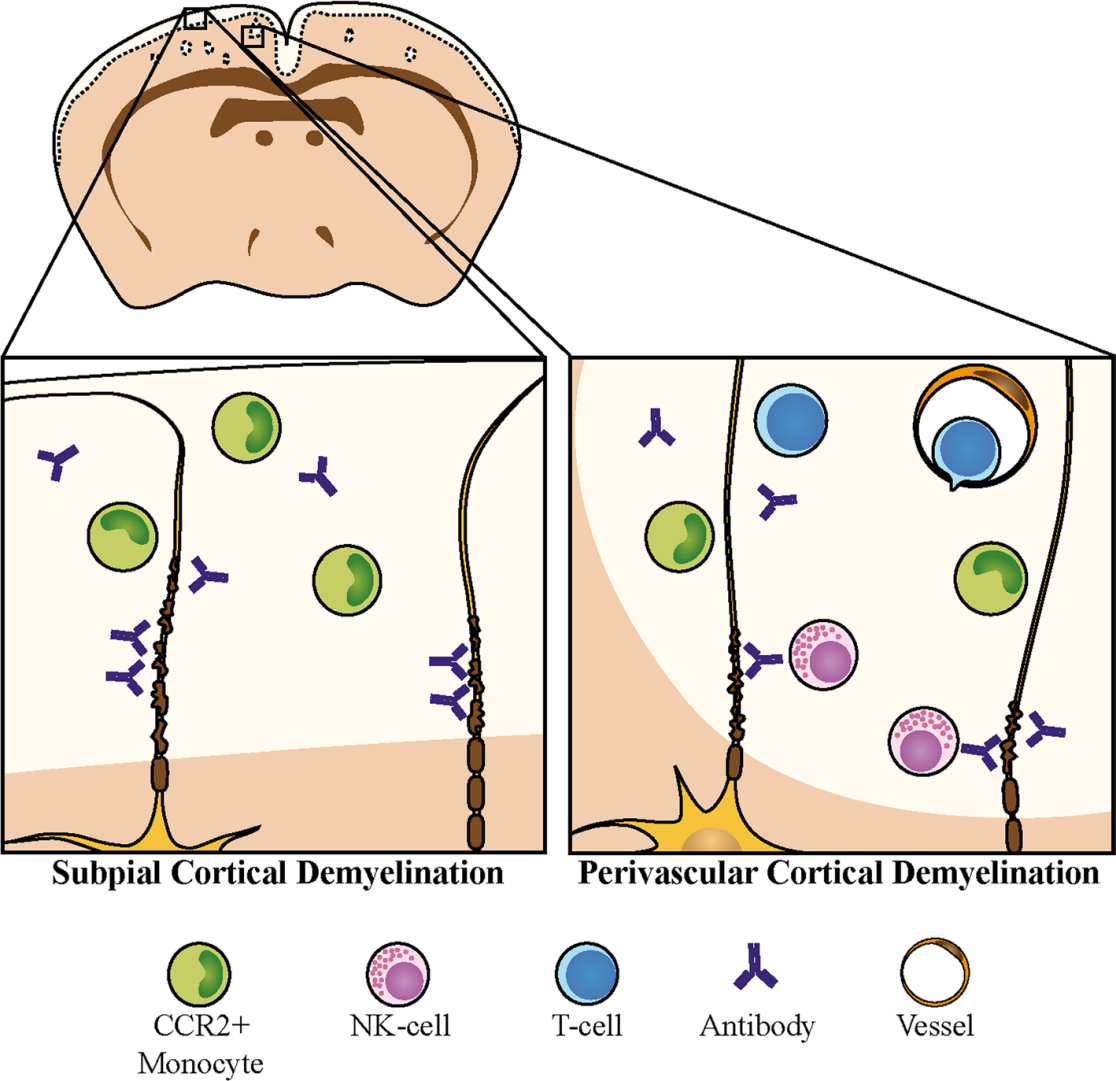
Differential involvement of immune effector mechanisms in perivascular type 2 and subpial type 3 cortical demyelination. Whereas subpial cortical demyelination occurs in the absence of an adaptive immune response, executed by the concerted action of pro-inflammatory cytokines, demyelinating antibodies, and CCR2^+^ monocytes, activated encephalitogenic T cells and NK cells substantially amplify perivascular cortical demyelination

The cerebral cortex is a major site of disease-related pathology in MS, whereby subpial cortical regions are the most frequently and intensely affected. We demonstrate here in biopsy tissue of MS patients that subpial lesion areas are more densely infiltrated by T cells and macrophages than deeper cortical lesion areas, supporting the idea that activated immune cell invasion primarily takes place via pial vessels. In addition, ongoing recruitment of CCR2^+^ monocytes accompanying active demyelination was observed, even in a patient with long-standing, progressive MS. Assessing the relevance of cellular and humoral immune mediators for cortical demyelination in an anti-myelin antibody-dependent experimental model, we show here that the requirements for immunological effector functions differ between subpial type 3 and perivascular type 2 cortical lesions (summarized in Fig. 9). Whereas cortical demyelination in both cortical lesion types was dependent on the presence of pathogenic demyelinating antibodies and inflammatory monocytes, the cellular requirements for perivascular cortical demyelination were more critical and included activated encephalitogenic T cells. Also, NK cells strongly aggravated the extent of perivascular cortical demyelination.

The less restrictive requirements for subpial cortical demyelination were corroborated by the observation that the intracortical injection of cytokines into healthy Th/+ mice readily increased the permeability of subpial but not of intracortical vessels to FITC-albumin. This is in line with the well-established increased permeability of human leptomeningeal endothelial cells compared to brain parenchymal endothelial cells and the reported stronger reactivity of meningeal blood vessels to pro-inflammatory cytokines [40]. Accordingly, the IgG leakage detected in an individual MS patient with active cortical demyelination was located subpially. Thus, distinct barrier properties within the cortical vasculature might facilitate the observed preferential subpial lesion localization in MS patients.

Interestingly, terminal complement activation is not the relevant antibody effector function for cortical demyelination in our animal model and its relevance for MS in general and for cortical demyelination in particular is discussed controversially. While Watkins and colleagues found increased numbers of cells, in particular neurons, in cortical gray matter lesions, which immunostained for the membrane attack complex (MAC) [52], Brink and colleagues found no evidence for complement activation in cortical MS lesions with evidence for microglia/macrophage activation [9]. In our biopsy cohort, terminal complement activation was not detectable in gray matter lesions even in actively demyelinating cortical lesions with subpial Ig leakage and/or ongoing monocyte recruitment. In line, the extent of cortical demyelination in Th/+ mice C1q^−/−^ and Th/+ mice C1q^+/+^ mice was comparable. Furthermore, the deficiency of complement regulatory proteins such as CD59a which block the assembly of the MAC on oligodendrocytes did not aggravate cortical demyelination in Th/+ mice. Cortical demyelination, however, was diminished by the locally applied BB5.1 antibody, which binds to C5 and thus prevents the generation of the cytolytic membrane attack complex by all three complement activation pathways. This suggests that the alternative complement activation pathway might also contribute to cortical demyelination as suggested by previous EAE studies for spinal cord pathology [24]. In summary, antibody-opsonization and ADCC are the more relevant effector functions of demyelinating antibodies in this model of inflammatory cortical demyelination.

In vivo evidence for NK cell-mediated ADCC has been provided in NMO animals with pathogenic AQP4 antibodies [43], while most MOG_35–55_-induced EAE studies focused on the immunoregulatory properties of NK cells [19]. Previous studies have also demonstrated that NK cells—among others via the activating receptor NKG2D—can kill oligodendrocytes in vitro independently of pathogenic antibodies [38, 46]. We detected perivascular NK cells in the demyelinated cortex of MS patients. In the presence of pathogenic antibodies, NK cells substantially contributed to the extent of perivascular, but not subpial demyelination in our mouse model. However, as expected, the extravasation of NK cells into the cortical parenchyma required activated encephalitogenic T cells.

Macrophages are the dominant inflammatory cell type in MS white matter lesions [10] and exert crucial effector functions in EAE [22]. Recent evidence has been provided that CCR2^rfp/+^-tagged monocyte-derived macrophages contribute to the initiation of demyelination at the node of Ranvier in spinal cord white matter EAE [53] and that Ly6C^high^ CCR2^+^ inflammatory monocytes licensed by GM-CSF are important for EAE induction [13]. The human orthologues of mouse inflammatory monocytes which might extravasate and differentiate into inflammatory macrophages in the CNS of MS patients are less well defined. We immunostained for CD14 and CCR2 which are expressed by classical monocytes in the bloodstream and found evidence both for CD14^+^ CCR2^+^ and CD14^+^ CCR2^−^ monocytes mainly perivascularly in the human demyelinated cortex. We interpreted these findings as a recruitment of monocytes into the inflamed cortex with CD14^+^ CCR2^−^ monocytes having either internalized CCR2 in response to ligand binding [34] or being recruited by additional chemokine receptor/ligand interactions [49]. The former explanation is supported by cytoplasmic CCR2 immunoreactivity in some of the invading monocytes. In line, CCR2^+^ cells colocalized with Iba-1^+^ intra- and perivascular monocytes, and less frequently with foamy and phagocytosing KiM1P+ cells. No relevant overlap between CCR2 and TMEM119 as well as P2Y_12_, recently described microglia markers, was observed in actively demyelinating MS lesions. Most importantly, perivascular monocytes, as well as T cells infiltrating cortical demyelinating lesions could be detected in patients with long-standing progressive MS. This suggests that invading immune cells are not only involved in the process of cortical demyelination in a cohort of MS patients with atypical or severe presentation early in the disease, but also in patients with chronic MS.

These findings encouraged us to develop a humanized anti-CCR2 antibody, which significantly reduced perivascular cortical demyelination and ameliorated the disease course in a preclinical model of non-human primate EAE, underscoring the relevance of CCR2^+^ monocytes for inflammatory cortical demyelination. Interestingly, we could not demonstrate a significant reduction of subpial cortical demyelination in CCR2^+^ monocyte-depleted marmosets, which might be due to very low animal numbers. Additionally, we might have initiated the depletion of CCR2^+^ monocytes too late and some of them might already have extravasated into the meninges. The expression of activated leukocyte cell adhesion molecule (ALCAM) which participates in the multi-step extravasation process of monocytes across the BBB, is significantly higher in human meningeal endothelial cells than in brain endothelial cells [25].

In considering the clinical translation of our experimental findings for the treatment of progressive MS, we acknowledge a number of limitations. We initiated the cortical demyelination in the Th/+ mouse by the intracortical injections of TNFα and IFNγ. Although both cytokines are increased in the CSF of MS patients with cortical demyelination, the inflammatory CSF milieu in MS is clearly more complex [14]. The mouse model, as well as the marmoset EAE model relied on the presence of demyelinating MOG-specific antibodies, which are rarely detected in MS patients [48]. Finally, the quite rapid resolution of cortical inflammation and demyelination seen in animal models even after multiple intracortical cytokine injections [44] and the relative lack of neuroaxonal damage are in contrast to the widespread and ongoing cortical demyelination and the extent of neuroaxonal loss seen in MS. Irrespective of the shortcomings of all currently available experimental models for MS, the success of a number of treatment strategies for the human disease, which were established first in animal models, such as, e.g., natalizumab [42, 54], demonstrate that shared and pathogenetically relevant mechanisms exist in our experimental models and the human disease.

In conclusion, cortical demyelination in general and subpial demyelination in particular are highly characteristic features of progressive MS. Our data indicate that distinct local barrier properties may underlie the differential tissue vulnerability in the cerebral cortex. Furthermore, we reveal that only a limited number of effector mechanisms are required for subpial demyelination in a novel mouse model of cortical inflammatory demyelination. The limited necessity for peripheral adaptive immune activation for the pathogenesis of subpial (type 3) demyelination may in part explain the difficulties encountered in therapeutically targeting the ongoing tissue damage in the progressive phase of the disease. Importantly, CCR2^+^ monocytes play a central role in the pathogenesis of cortical demyelination and their depletion proves a novel therapeutic concept to ameliorate cortical pathology.

## Electronic supplementary material

Below is the link to the electronic supplementary material.

Supplementary material 1 (TIFF 702 kb)

Supplementary material 2 (TIFF 28322 kb)

Supplementary material 3 (TIFF 6576 kb)

Supplementary material 4 (TIFF 14393 kb)

Supplementary material 5 (TIFF 11671 kb)

Supplementary material 6 (TIFF 28423 kb)

Supplementary material 7 (DOCX 19 kb)

Supplementary material 8 (DOCX 12 kb)
