# Supplementary figures and images for: Differential contribution of immune effector mechanisms to cortical demyelination in multiple sclerosis
Source: Acta Neuropathol. 2017 Apr 6;134(1):15–34. doi: 10.1007/s00401-017-1706-x (PMC5486638; doi:10.1007/s00401-017-1706-x)

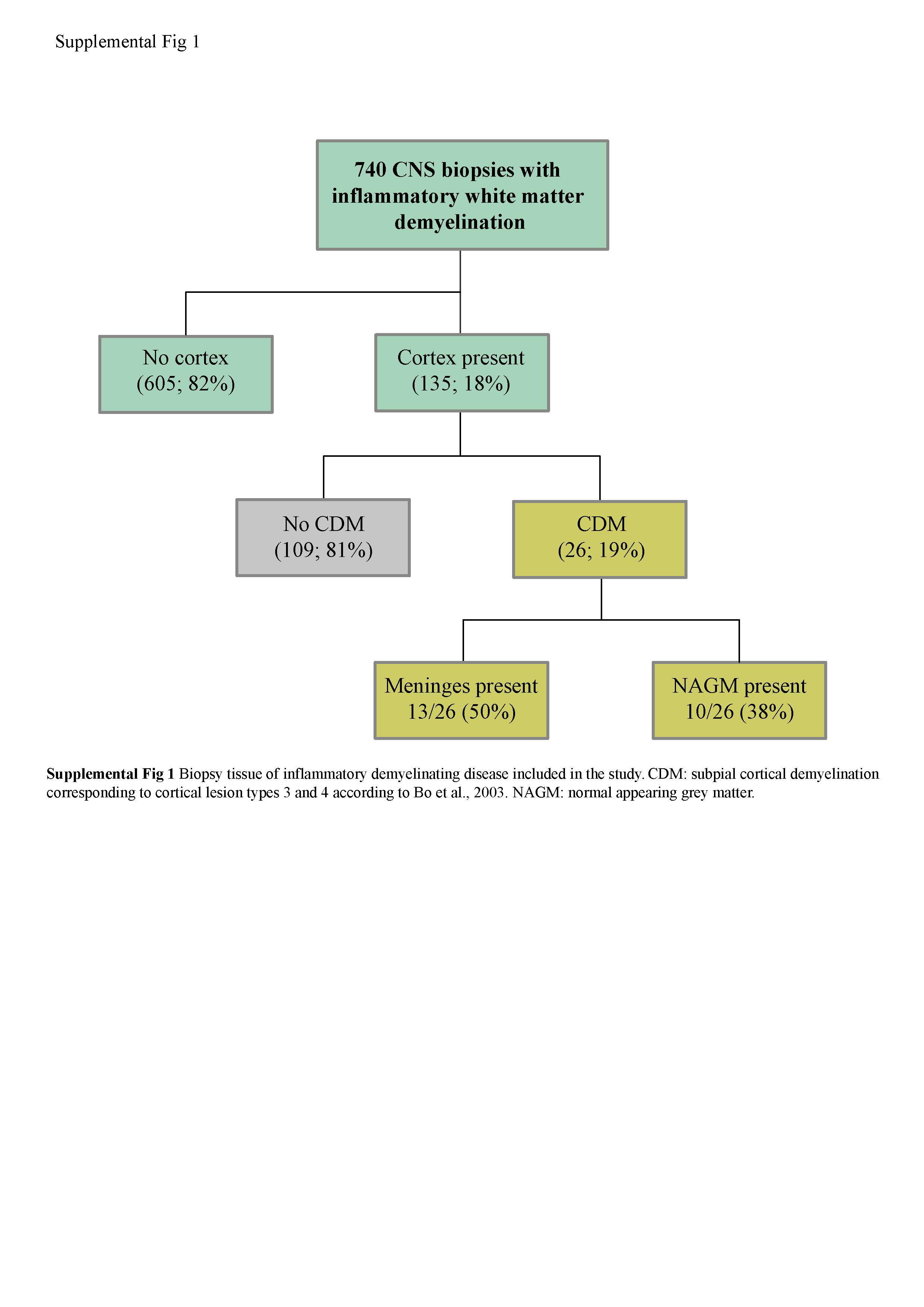

Supplement: Supplementary file 1 — Supplementary material 1 (TIFF 702 kb) [file 401_2017_1706_MOESM1_ESM.tif]

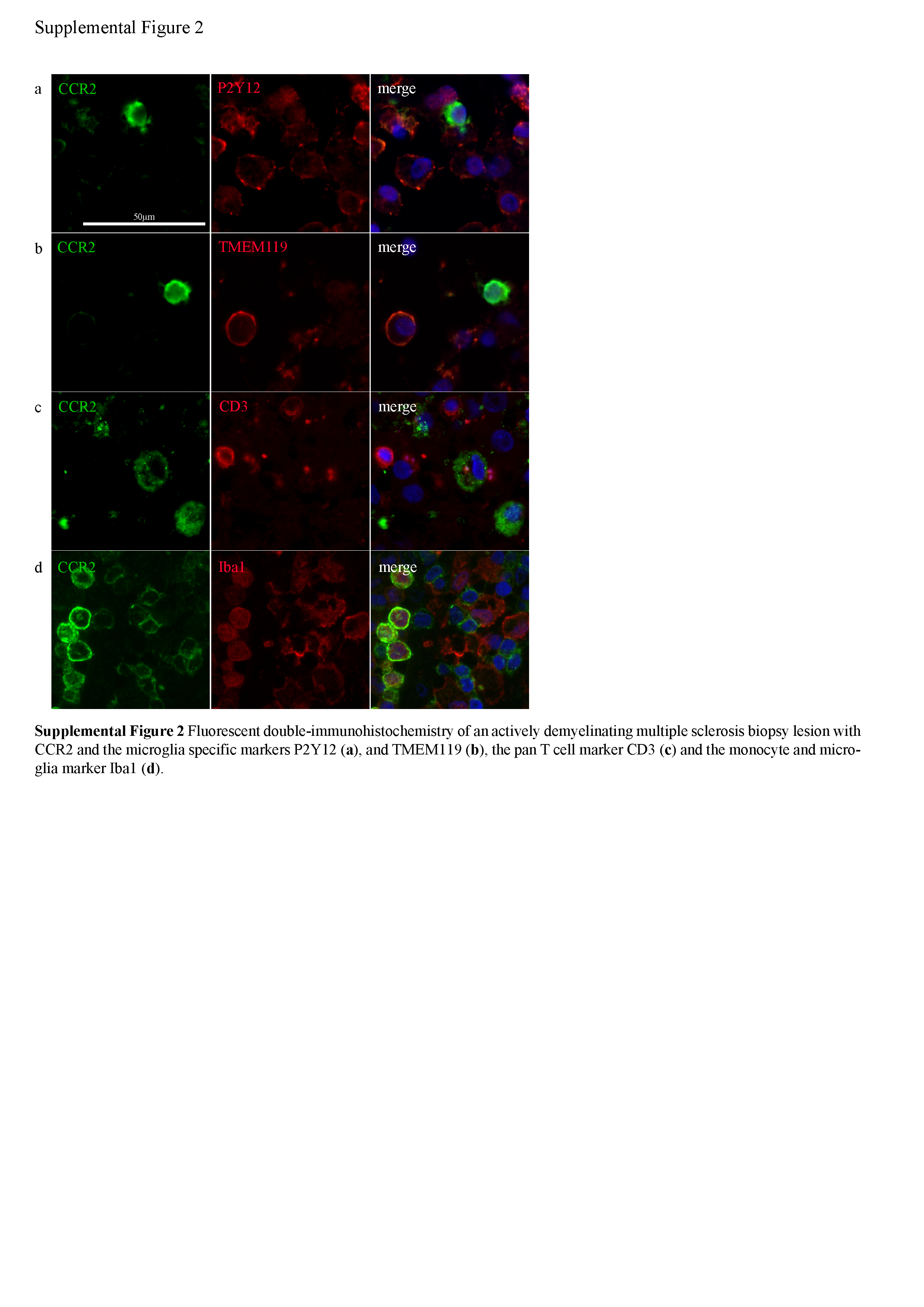

Supplement: Supplementary file 2 — Supplementary material 2 (TIFF 28322 kb) [file 401_2017_1706_MOESM2_ESM.tif]

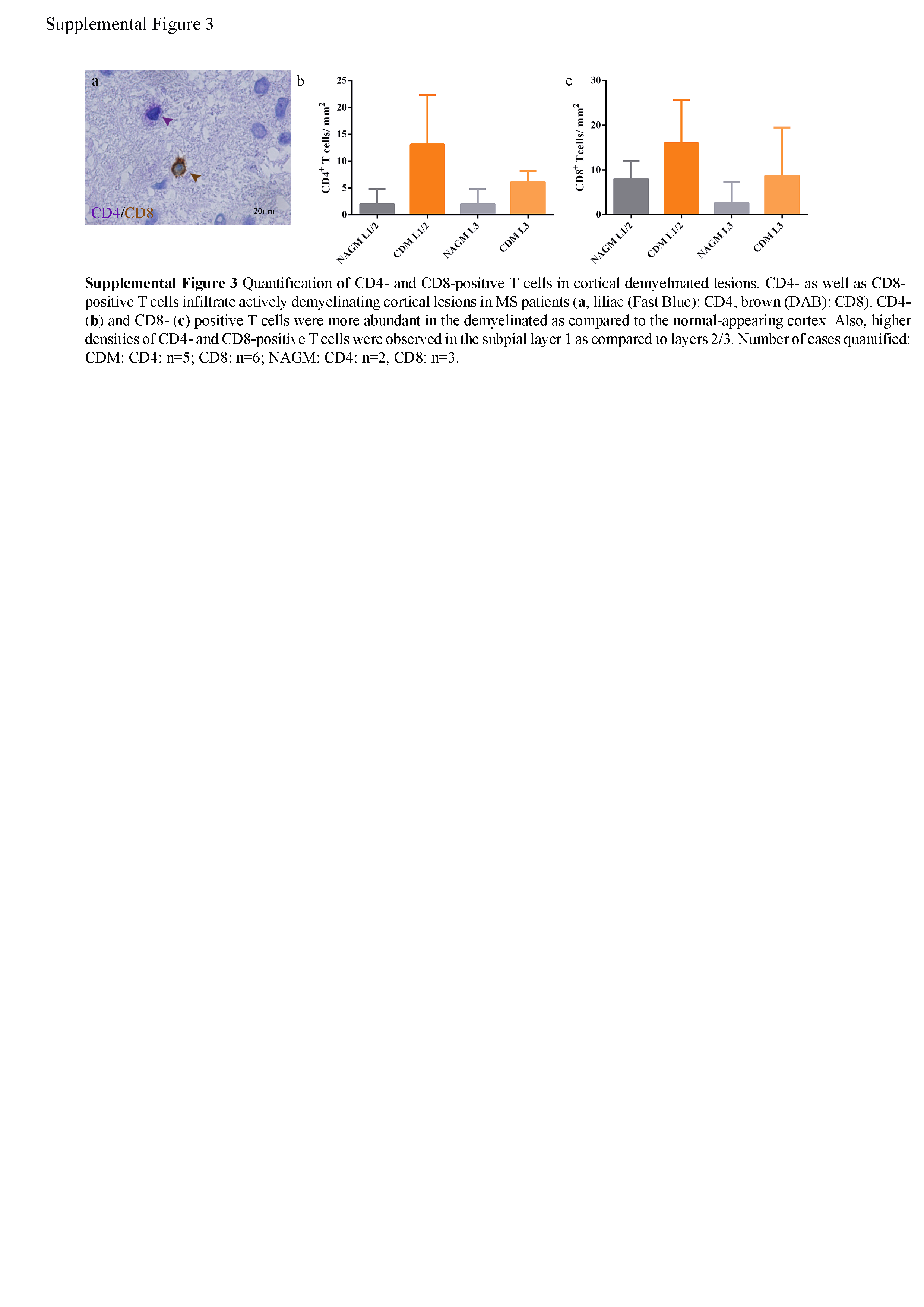

Supplement: Supplementary file 3 — Supplementary material 3 (TIFF 6576 kb) [file 401_2017_1706_MOESM3_ESM.tif]

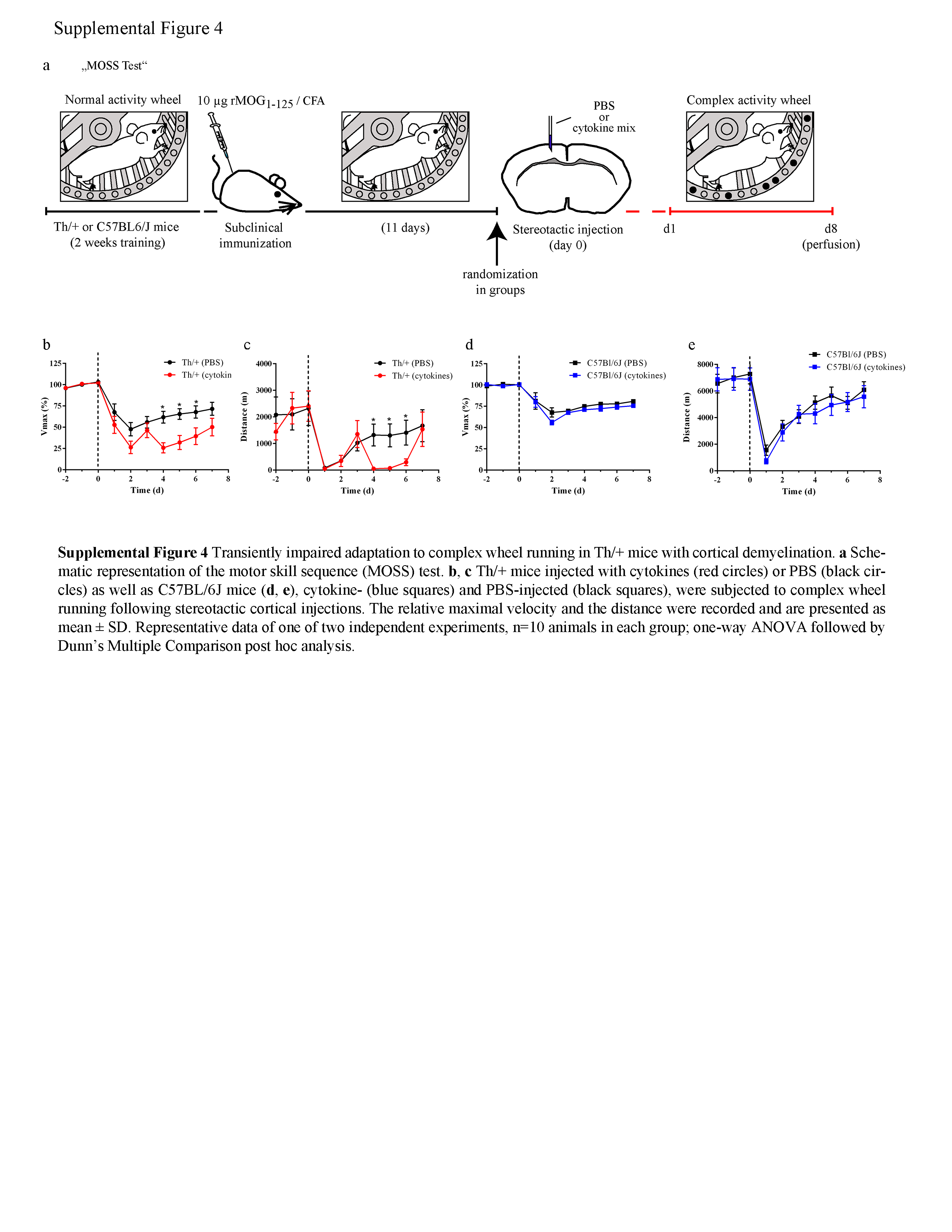

Supplement: Supplementary file 4 — Supplementary material 4 (TIFF 14393 kb) [file 401_2017_1706_MOESM4_ESM.tif]

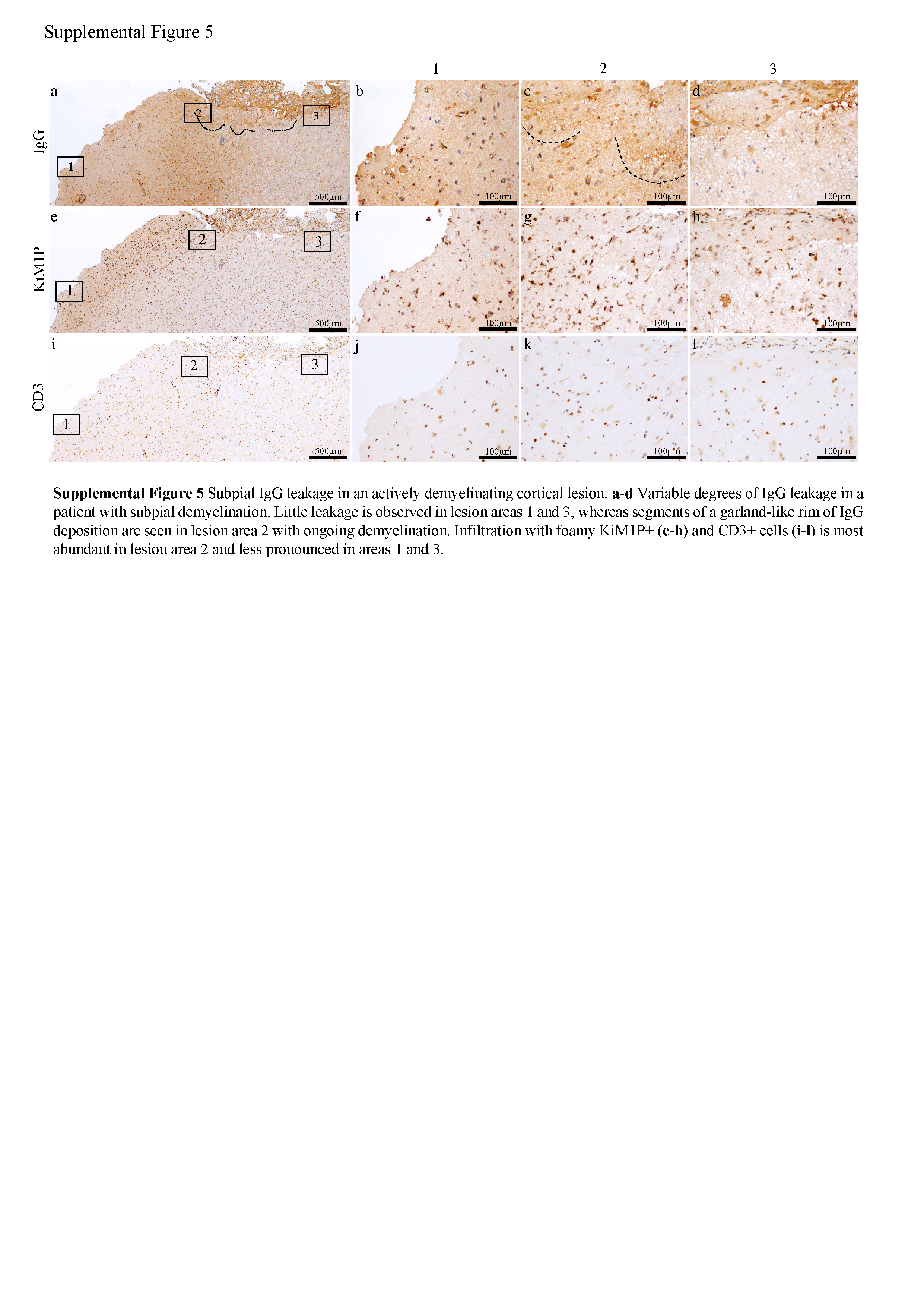

Supplement: Supplementary file 5 — Supplementary material 5 (TIFF 11671 kb) [file 401_2017_1706_MOESM5_ESM.tif]

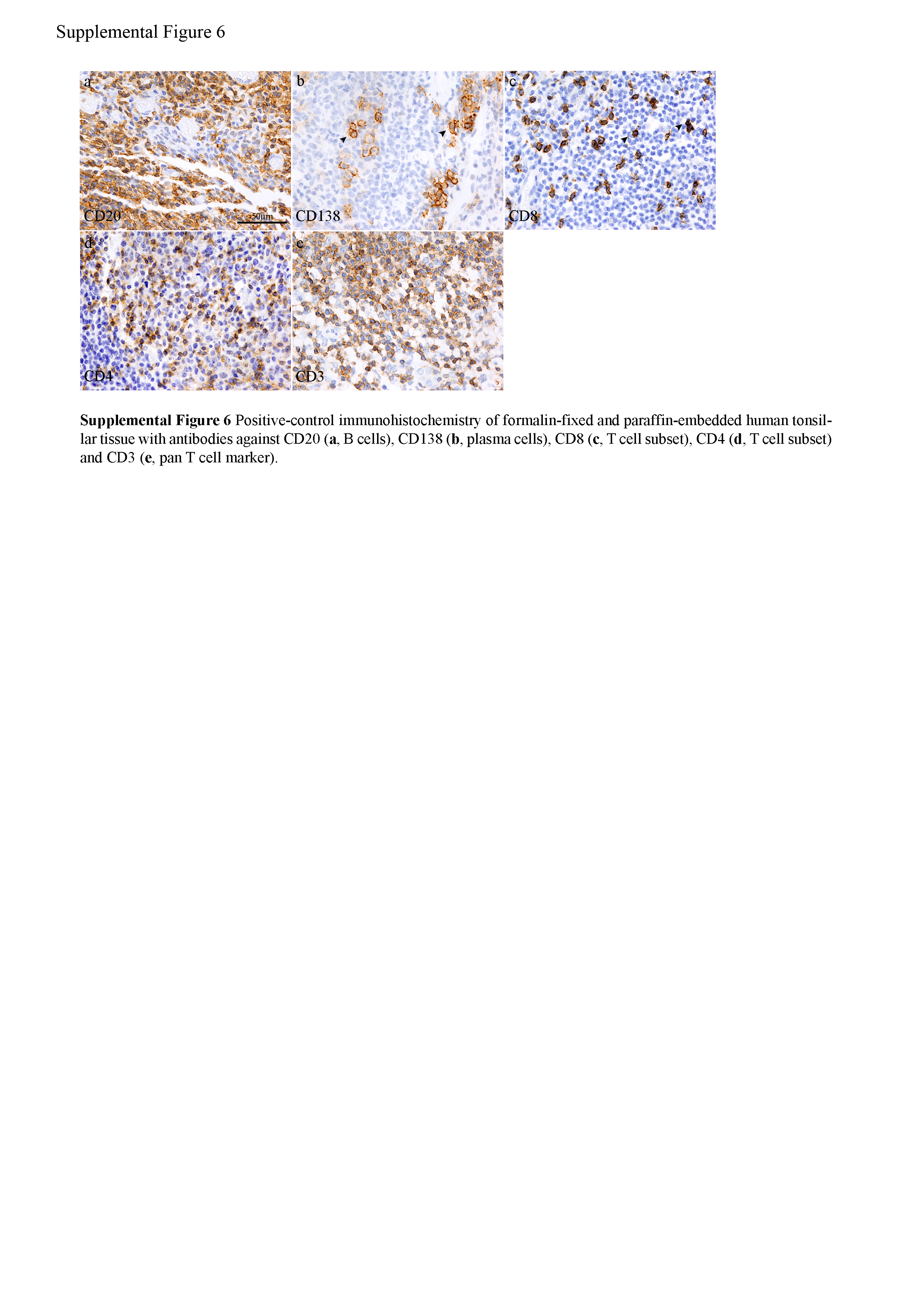

Supplement: Supplementary file 6 — Supplementary material 6 (TIFF 28423 kb) [file 401_2017_1706_MOESM6_ESM.tif]
