# Supplementary material for: Differential contribution of immune effector mechanisms to cortical demyelination in multiple sclerosis
Source: Acta Neuropathol. 2017 Apr 6;134(1):15–34. doi: 10.1007/s00401-017-1706-x (PMC5486638; doi:10.1007/s00401-017-1706-x)
Supplement: Supplementary file 7 — Supplementary material 7 (DOCX 19 kb) [file 401_2017_1706_MOESM7_ESM.docx]

**Supplemental Table 1**

| **MS**  **case** | **Age/**  **Sex** | **Disease**  **duration**  **(months)** | **Disease**  **course** | **Presenting**  **symptom(s)** | **Brain MRI** | **Index lesion** |
| --- | --- | --- | --- | --- | --- | --- |
| #1 | 45/F | 6 | CIS | Slight apraxia and unsteady gait | Multiple periventricular and subcortical WM lesions; cortical lesions | Right frontal |
| #2 | 49/F | 11 | RRMS | Blurred vision of the right eye | Multiple WM lesions; subcortical, cortical and brainstem lesions | Left parietal |
| #3 | 34/F | 3 | RRMS | Blurred vision of the left eye | Multiple WM lesions; juxtacortical and brainstem lesions, in part contrast-enhancing | Right temporal |
| #4 | 26/F | 2 | CIS | Minor weakness of the left hand, symptoma-tic epilepsy with focal motor seizures | Several intracerebral WM lesions; three subcortical lesions partly ring-enhancing; two brainstem lesions | Right frontal |
| #5 | 13/F | 116 | RRMS | Weakness of both legs, pre-existing sympto-matic epilepsy, hand tremor, dysarthria | Multiple, partly contrast-enhancing WM lesions | Left frontal |
| #6 | 42/F | 2 | CIS | Vision loss, disturbed balance, depression, beginning dementia | Multiple subcortical and periventricular WM lesions, in part contrast-enhancing | Left frontal |
| #7 | 64/M | 1 | CIS | Fatigue, aphasia | Three lesions precentral left, temporal left, frontal right, partly contrast-enhancing | Left temporal |
| #8 | 49/M | 228 | RRMS | Symptomatic epilepsy, hemianopsia to the left | Right occipital space-occupying lesion with weak contrast enhancement of the lesion borders. | Right occipital |
| #9 | 50/F | ~2 | CIS | Paresis and paresthesia of the right leg | Tumefactive left frontal lesion without contrast enhancement. | Left frontal |
| #10 | 51/F | 0·3 | CIS | Headache and vomiting | Singular right frontal contrast-enhancing lesion with perifocal oedema | Right frontal |
| #11 | 33/M | 0·5 | CIS | Decreased vision right eye | Multiple contrast enhancing cerebral WM lesions with perifocal oedema. | Right frontal |
| #12 | 49/M | 0·3 | CIS | Left hemiparesis, confusion | Single, tumefactive cystic right frontal lesion with contrast-enhancing borders | Right frontal |
| #13 | 32/M | N/A | CIS | Symptomatic epilepsy, left hemianopsia, confusion | N/A | Cingulate gyrus |
| #14 | 48/F | 216 | SPMS | Left arm weakness, left-accentuated spastic paraparesis, myoclonus | Multiple WM lesions; two contrast-enhancing cortico-subcortical lesions, leptomeningeal enhancement; cerebellar and brainstem lesions | N/A |
| #15 | 28/F | 0·5 | CIS | Numbness and weakness of the right hand, aphasia | Singular left parietal contrast-enhancing lesion with perifocal oedema | Left parietal |
| #16 | 72/M | 1 | CIS | Progressive gait ataxia, spastic paraparesis | T2 hyperintense lesions in the right frontal lobe and brainstem | Right frontal |
| #17 | 38/F | 120 | RRMS | Symptomatic epilepsy, anomic aphasia, ataxia, right-sided spastic hemiparesis | Multiple periventricular and corpus callosum T2 hyperintense lesions; left frontal contrast-enhancing lesion with perifocal oedema. | Left frontal |
| #18 | 42/M | 0·5 | CIS | Acute gait disturbance, blurred vision of the left eye, right hemiparesis | Left temporal WM lesion; lesion in the right cerebellar peduncle; right frontal cortical lesion, partial contrast enhancement | Left temporal |
| #19 | 56/F | 204 | SPMS | Symptomatic epilepsy, dementia, spastic left and leg accentuated tetraparesis | Multiple periventricular lesions, brainstem lesions, partly contrast-enhancing | Left frontal |
| #20 | 40/F | 3 | CIS | Spastic paraparesis, confusion | Multiple WM lesions in the corpus callosum; basal ganglia lesions, partially contrast-enhancing | Right frontal |
| #21 | 20/M | 1 | CIS | Left hemiparesis | Right parietal WM lesion with perifocal oedema and mild contrast enhancement | Right parietal |
| #22 | 62/F | 0·2 | RRMS | Symptomatic epilepsy, aphasia, right-sided hemiparesis | Single tumefactive left temporo-parietal contrast-enhancing lesion with perifocal oedema. | Left parietal |
| #23 | 19/F | 0·5 | CIS | Quadrantanopsia to the left, dysphagia, headache, depression | Right tumefactive parieto-occipital lesion, multiple contrast-enhancing lesions in the right cerebellar lobule and vermis. | Right parieto-occipital |
| #24 | 32/F | 24 | RRMS | Symptomatic epilepsy, myoclonus of the left arm, left hemiparesis | Multiple WM lesions; contrast-enhancing cortical lesions | N/A |
| #25 | 31/F | 24 | RRMS | Right hemiparesis, cognitive impairment | Multiple ring-enhancing WM lesions | Left parietal |
| #26 | 27/F | 0·8 | CIS | Right hemiparesis | Single left ring-enhancing parietal lesion | Left frontal |

**Table S1. Clinical data of patients included in the study.**
