# Supplementary material for: Differential contribution of immune effector mechanisms to cortical demyelination in multiple sclerosis
Source: Acta Neuropathol. 2017 Apr 6;134(1):15–34. doi: 10.1007/s00401-017-1706-x (PMC5486638; doi:10.1007/s00401-017-1706-x)
Supplement: Supplementary file 8 — Supplementary material 8 (DOCX 12 kb) [file 401_2017_1706_MOESM8_ESM.docx]

**Supplemental Table 2**

| **Genotype** | **n** | **Disease onset**  **mean +/- SD** | ***P* value** | **Maximum score**  **mean +/- SD** | ***P* value** |
| --- | --- | --- | --- | --- | --- |
| Th/+ CCR2^+/+^ | 13 | 11.2 +/- 1.1 | - | 3.3 +/- 0.5 | - |
| Th/+ CCR2^-/-^ | 5 | 15 +/- 1 | < 0.001  (unpaired t-test) | 3.2 +/- 0.3 | NS  (MWU) |

**Table S2. Statistical analysis of clinical EAE in Th/+ CCR2^+/+^ vs Th/+ CCR2^-/-^ mice.**
